# Significance of root hairs at the field scale – modelling root water and phosphorus uptake under different field conditions

**DOI:** 10.1007/s11104-019-04308-2

**Published:** 2019-12-06

**Authors:** S. Ruiz, N. Koebernick, S. Duncan, D. McKay Fletcher, C. Scotson, A. Boghi, M. Marin, A. G. Bengough, T. S. George, L. K. Brown, P. D. Hallett, T. Roose

**Affiliations:** 1 Bioengineering Science Research Group, Department of Mechanical Engineering, School of Engineering, Faculty of Engineering and Physical Science, University of Southampton, Southampton, SO17 1BJ UK; 2 School of Biological Sciences, University of Aberdeen, Aberdeen, AB24 3UU UK; 3 Ecological Sciences Group, The James Hutton Institute, Invergowrie, Dundee, DD2 5DA UK; 4 School of Science and Engineering, University of Dundee, Dundee, DD1 4HN UK; 5 Institute of Agricultural and Nutritional Sciences, Martin Luther University Halle-Wittenberg, Universitaetplatz 10, 06108 Halle (Saale), Germany

**Keywords:** Mathematical modelling, Plant roots, Rhizosphere, Soil, Water, Phosphorus, Root hairs, Field

## Abstract

**Abstract:**

**Background and aims:**

Root hairs play a significant role in phosphorus (P) extraction at the pore scale. However, their importance at the field scale remains poorly understood.

**Methods:**

This study uses a continuum model to explore the impact of root hairs on the large-scale uptake of P, comparing root hair influence under different agricultural scenarios. High vs low and constant vs decaying P concentrations down the soil profile are considered, along with early vs late precipitation scenarios.

**Results:**

Simulation results suggest root hairs accounted for 50% of total P uptake by plants. Furthermore, a delayed initiation time of precipitation potentially limits the P uptake rate by over 50% depending on the growth period. Despite the large differences in the uptake rate, changes in the soil P concentration in the domain due to root solute uptake remains marginal when considering a single growth season. However, over the duration of 6 years, simulation results showed that noticeable differences arise over time.

**Conclusion:**

Root hairs are critical to P capture, with uptake efficiency potentially enhanced by coordinating irrigation with P application during earlier growth stages of crops.

**Electronic supplementary material:**

The online version of this article (10.1007/s11104-019-04308-2) contains supplementary material, which is available to authorized users.

## Introduction

Phosphorus (P) is an essential element for plant growth and reproduction and is therefore critical for maximizing crop yield . However,poor management of P has severe environmental consequences (Cordell et al. 2009; Dawson and Hilton 2011; Sylvester-Bradley et al. 2017). An understanding of the biophysical mechanisms associated with the transport of P in the root zone (known as the rhizosphere (Daly et al. 2017)) aids the development of more sustainable agricultural practices that use and lose less P (Heppell et al. 2016). Inherent difficulties arise when quantifying root-soil interactions, as their spatial relevance spans from the size of an individual soil pore up to an entire field (Vereecken et al. 2016). Plant roots further complicate the problem by perturbing soil physical, chemical, and biological properties in the rhizosphere at the interface between the root and the soil (Koebernick et al. 2017). In the context of feeding a growing population (Vereecken et al. 2016), methods are needed to more effectively assess the influence of rhizosphere processes at the field scale. Therefore, it is important to untangle root biophysical interactions at soil pore scale from the transport of water and nutrients in the field to determine the most important mechanisms driving phosphorus uptake.

Individual plant roots grow through soil following beneficial mechanical and moisture gradients in order to obtain water and nutrients (Colombi et al. 2017; Eapen et al. 2005). By applying localized suction around the root-soil interface, plant roots pull water towards them (Duncan et al. 2018). Root exudates could alter the mass flow of nutrients as they are known to augment water retention and transport processes (Ahmed et al. 2014; Kroener et al. 2014), stimulate synergistic micro fauna (Kuzyakov and Blagodatskaya 2015), temporarily reduce soil impedance during growth (Bengough and Mullins 1990; Naveed et al. 2017), and even dissolve less-mobile nutrients (Masaoka et al. 1993). Another key method allowing plants to extract resources from the soil is their ability to produce root hairs (Koebernick et al. 2017). Root hairs assist with root growth through soil by means of anchoring (Bengough et al. 2016), and possibly modify the rhizosphere allowing the root to acquire less readily accessible P reserves (Brown et al. 2012a; Haling et al. 2013; Jones 1998). Root hairs may also contribute to water uptake by extending the zone of soil root influence (Segal et al. 2008), which is further facilitated by reducing the gradients in matric potential near the root soil interface (Carminati et al. 2017).

Root hairs have shown particular efficacy for extracting P from soil (Bates and Lynch 2001; Haling et al. 2013; Keyes et al. 2013) as they increase the effective surface area of plant roots (Silberbush and Barber 1983). Phosphorus is an anion that binds to positively charged exchange sites on fine textured soil particles (Turner et al. 2005), limiting its effective mobility in soil (Aharoni and Sparks 1991). This limited mobility of P keeps it in the surface layers of soil, so shallow plant roots with good root-soil contact optimize extraction of P. Root hair proliferation allows roots to exploit otherwise non-accessible stocks of P (Haling et al. 2013), likely by increasing the root-soil contact and having access to finer pores than the main root axis can enter (Tisdall 1991). Physical characteristics of root hairs such as length and root hair densities have shown to be significant in nutrient acquisition (Foehse and Jungk 1983). Increased root hair lengths have shown to enhance zones of nutrient depletion via enhanced nutrient uptake (Ma et al. 2001). Increased root hair densities have shown to be particularly beneficial for root uptake for nutrients with low diffusivities (Ma et al. 2001). As a consequence, the quantities of P taken up by root hairs can be significant, reaching quantities almost equivalent to the those taken up by the rest of the plant root system (Keyes et al. 2013).

Previous model studies have tried to assess the impacts that root hairs have on general root uptake processes. This includes root hair lengths and densities, which have been modelled to have a large impact on the overall bulk nutrient uptake (Brown et al. 2012a; Ma et al. 2001). More physically explicit models exploring the impact that root hairs have on enhancing the proximity of roots to nutrients (Itoh and Barber 1983; Leitner et al. 2010) have found that root hairs could increase the radial zone of influence of roots by up to 0.4 mm. Direct quantification of root hairs and soil pore space by image based modelling at the rhizosphere scale under different moisture conditions (Daly et al. 2016) suggests root hair development may be very dynamic, ultimately accounting for nearly 50% of the total uptake flux. Recent models have also proposed that root hairs may buffer the soil potential flux close to the root surface caused by transpiration, maintaining wetter moisture regimes local to the root (Carminati et al. 2017).

While root hairs play an important role in P extraction at the pore scale, it is not clear to what degree this extends to the field scale. More importantly, there exist a number of environmental factors whose interactions can heavily influence P uptake by roots from soil. For example, agricultural treatments such as tillage can alter how nutrients are distributed along the soil depth (Steiner et al. 2007). Agricultural treatments will also have a large impact on root architecture, with plants growing in compacted soils being constrained. Furthermore, precipitation patterns will impact soil moisture, which influences how efficiently roots are able to extract P from the soil. For this purpose, our aim is to use modelling approaches to systematically explore how different environmental conditions and root hairs impact root water and P uptake. Specifically, our objectives are to:

1. Use the pore scale influence of root hairs to estimate their impact in a field scale model;
2. Consider the effect of different concentrations of P in the soil;
3. Simulate the impact of different but comparable distributions of P down the soil profile; and
4. Assess the effect that different simulated precipitation patterns have on P uptake.

We use a combined water and solute transport model (Heppell et al. 2016; Roose and Fowler 2004a) to evaluate how P location, P availability and precipitation for soils under different soil management treatments influences the impact root hairs on P uptake. Simulations are extended to multiple growing seasons that use real sets of rainfall data to explore the impact on the final soil P-stocks. Finally, we discuss the results in the broader context of plant breeding and agricultural practices.

## Theoretical considerations

### Overview of the significance to root hairs in P acquisition

Root hairs are formed from either individual or a string of thin tube-like cells that grow outward perpendicular to the root epidermal surface (Brown et al. 2012b). While some studies report that root hairs assist in root water uptake (Carminati et al. 2017), root hairs are thought to develop primarily for nutrient uptake (Brown et al. 2012b). As roots develop hairs, they increase the root’s surface area, accounting for up to 70% of the total root area (Raghothama and Karthikeyan 2005). This acts to maximize P absorption potential (Brown et al. 2012b; Silberbush and Barber 1983). The geometric scale of root hairs (approximate diameter of 10 μm) enables them to exploit scarce P stocks otherwise inaccessible to the plant due to their immobility (Tisdall 1991). Furthermore, root hairs entangle and adhere to soil, creating an outer layer of aggregates known as the rhizosheath (Delhaize et al. 2009; Koebernick et al. 2017). The rhizosheath facilitates root P uptake via enhanced diffusion towards the root by reducing the tortuosity of diffusion pathways (Brown et al. 2012b; Brown et al. 2017; Pang et al. 2017). The rhizosheath forms a cluster of soil aggregates, which is abundant in microbes and mycorrhizal fungi (George et al. 2014), further interacting with the plant root and facilitate root P acquisition (Jakobsen et al. 2005). The combined effect results in root hairs accounting for over half of the total P taken up by the roots (Brown et al. 2012b; Keyes et al. 2013). Although there are many complex processes associated with enhanced P acquisition due to root hairs, the focus of this study is to assess the effect of root hairs with variation in environmental conditions at a larger scale. Therefore, this study assumes that root hairs account for half of the total P root uptake rate. Details regarding the model implementation are elaborated in the materials and methods section.

## Materials and methods

### Overview of modelling strategy

The modelling carried out in this study focused on barley (*Hordeum vulgare* L.) root growth in soil. The simulations complement previous experimental studies that investigated the differences in plant development by comparing root hair bearing wild type plants (hairy) to mutant lines with heavily suppressed root hair growth (hairless) (Brown et al. 2012b). The experiments in the study also considered limitations of P in the soil, as well as the influence of water shortage (Brown et al. 2012b).

Our modelling coupled root water and P uptake from the soil, considering variable precipitation scenarios and different P concentrations and distributions. This allows the model to implicitly consider the impacts of P limited soils and drought. The different scenarios carried out in this study are outlined in further detail in the Materials and Methods section. Simulations were conducted using the finite element software package COMSOL 5.3 Multiphysics (COMSOL, Inc., Stalkholm, Sweden)(Multiphysics 2015) using the general form PDE interface, and results were analysed using Matlab 2016 (Mathworks, Inc., Natick, MA, USA)(Guide 1998). A list of symbols and variables can be found in Table S1.

In this initial step to model root hair impacts on P uptake at field scale, several assumptions are made that are either valid for the field experiment being simulated, or provide scope for development in future studies. P loss from overland flow and run-off has been omitted as the soil studied was freely draining and experience low intensity rainfall, although it could have a small impact on P dynamics. Moreover, as P solubility is low, downward flux outside of the root zone is not considered, and evaporation effects on upward flux have not been considered as precipitation greatly exceeds evaporation.

### Modelling water flow through unsaturated soil

In order to consider a range of moisture scenarios and water exchange dynamics between soil and plants (Roose and Fowler 2004b), simulations are designed considering partially saturated soil conditions typical for most agricultural scenarios (Kirkham 2014). Water flow through partially saturated soils is a nonlinear process, as water moves predominately through micropores (Hoogland et al. 2015; Lehmann et al. 2017; Richards 1931). This becomes particularly crucial for plant roots under dryer conditions, as roots have to apply suctions exceeding the matric potential of the soil to extract water (Gardner 1960). We adopt a field scale model, originally developed by Roose and Fowler (2004b), that considers both water and nutrient movement in soil under the influence of root water uptake by plant roots. Modelling parameters are based on a sandy loam soil (Liang et al. 2017) (see later section on soil properties for details). Relevant modelling parameters were based on experimental evidence from this soil and are listed in Table 1. A detailed list of variable symbols can be found in Table S3.

**Table 1:** List of parameters used for modelling. Parameters are based on sandy loam

Parameter	Value	Units	Description
*a*_0_	5×10^−4^	[m]	Primary root radius
*a*_1_	2×10^−4^	[m]	Lateral root radius
*b*	239	[mol_dis_ mol^−1^_ads_]	Phosphate buffer power in soil
*d*	2	[−]	Tortuosity factor
*d*_*E*_	27.4×10^−6^	[m]	Diameter of early metaxylem elements
*d*_*L*_	92.3×10^−6^	[m]	Diameter of late metaxylem elements
*D*_0_	6.16×10^−8^	[m^2^ s^−1^]	Water diffusivity in soil
*D*_*f*_	1.16×10^−10^	[m^2^ s^−1^]	Phosphate diffusivity in water
*F*_*m*_	3.26×10^−8^	[mol m^−2^ s^−1^]	Max P uptake rate
*k*_*r*_	2.15×10^−13^	[m Pa^−1^ s^−1^]	Root cortical water conductivity
*k*_*z*_	1.35×10^−14^	[m^4^ Pa^−1^ s^−1^]	Root axial water conductivity
*l*_0, *f*_	0.5	[m]	Max primary root length
*l*_1, *f*_	8×10^−2^	[m]	Max lateral root length
*K*_*m*_	5.80×10^−3^	[mol m^−3^]	Michaelis-Menten parameter for P
*K*_*s*_	2.157×10^−7^	[m s^−1^]	Saturated hydraulic conductivity
*l*_0, 0_	5×10^−3^	[m]	Initial root length
*l*_0, *a*_	5×10^−2^	[m]	Branching depth
*l*_1, *n*_	2.5×10^−3^	[m]	Inter-nodal-distance of lateral roots for barley
*m*	0.3	[−]	Van-Genuchten pore size distribution parameter
*n*_*E*_	16.2	[−]	Number of early metaxylem elements
*n*_*L*_	6.6	[−]	Number of late metaxylem elements
*P*	-3×10^5^	[Pa]	Absolute value of root potential at the shoot
$\tilde{p}_{c}\left(=\rho g/\alpha \right)$	−6.5×10^3^	[Pa]	Soil characteristic potetential
*r*_0_	2×10^−7^	[m s^−1^]	Maximum root elongation rate
*α*	1.5	[m^−1^]	Van-Genuchten inverse air entry parameter
*β*	1.07	[rad]	Branching angle
*μ*	8.9×10^−4^	[Pa s]	Dynamic viscosity of water at 25^o^ C
*θ*_*s*_	0.32	[m^3^_water_ m^−3^_bulk_]	Water content at zero tension
*ρ*	997	[kg m^−3^]	Density of water at 25^o^ C
*θ*_*r*_	0.14	[m^3^_water_ m^−3^_bulk_]	Soil residual water content at wilting point

**Fig. 1:**
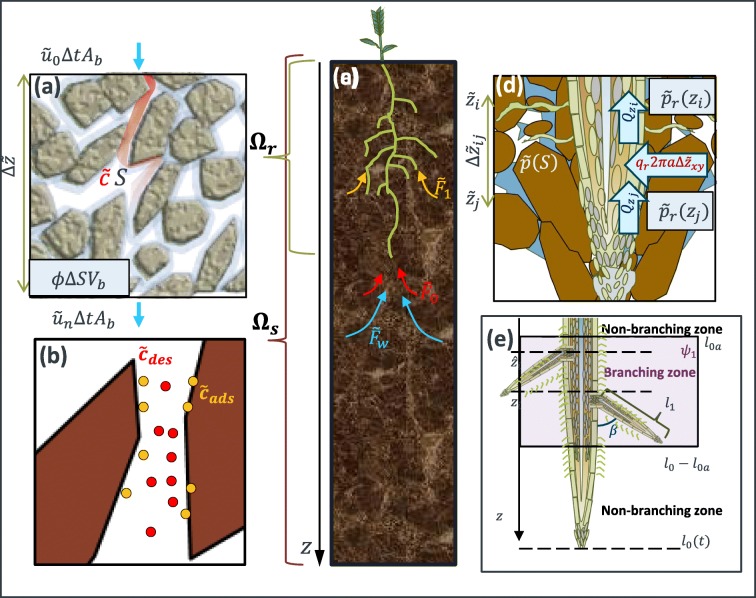
Conceptual model of water and solute movement through unsaturated soil with uptake from a growing plant root: Richards’ equation coupled with solute transport (**a**), are used to quantify the bulk scale movement and concentrations of water and solutes. The solute buffer power is considered (**b**) as a mechanism quantifying the adsorbed and desorbed nutrients. Simulations are run at the bulk scale (**c**) explicitly considering the effects of a growing plant through the 1D domain (Ω_*s*_ extending to 1 m depth)*,* where the water and solutes are taken up to the plant subdomain (Ω_*r*_). Mechanisms for plant water uptake are considered based on the water mass balance passing through the soil and up the xylem (**d**), and the explicit geometry of the branching roots is also considered (**e**). We highlight the distinction between lateral branching roots consisting of vascular structures from single cellular root hairs

Given a bulk soil domain, we model changes in soil moisture proportional to the inward and outward water fluxes (Fig. 1(a)) and the root water uptake (Fig. 1(c)) using a Richards’ type formulation (Richards 1931):

1 $$ \phi \frac{\partial S}{\partial \overset{\sim }{t}}+\overset{\sim }{\nabla}\cdot \overset{\sim }{\boldsymbol{u}}=-{\overset{\sim }{F}}_w,\kern0.5em \overset{\sim }{z}\in {\Omega}_{\mathrm{s}}, $$

where *ϕ* = (*θ*_*s*_ − *θ*_*r*_) [m^3^_pore_ m^−3^_bulk_] is the difference between the soil residual water content at wilting point (*θ*_*r*_ [m^3^_water_ m^−3^_bulk_]) and the soil water content at zero tension (*θ*_*s*_ [m^3^_water_ m^−3^_bulk_]), *S* [−] is the relative water saturation defined as *S* = (*θ*_*v*_ − *θ*_*r*_)/*ϕ*, where *θ*_*v*_ [m^3^_water_ m^−3^_bulk_] is the soils volumetric water content, $\overset{\sim }{\boldsymbol{u}}$ [m^3^_water_ m^−2^_bulk_ s^−1^] is the volumetric water flux, and ${\overset{\sim }{F}}_w$ [m^3^_water_ m^−3^_bulk_ s^−1^] is the uptake of water by plant roots in the bulk soil domain Ω_s_.

The volumetric water flux is defined by Buckingham-Darcy equation (Liu 2017):

2 $$ \overset{\sim }{\boldsymbol{u}}=-\frac{k}{\mu}\left(\overset{\sim }{\nabla}\overset{\sim }{p}-\rho g\hat{\boldsymbol{k}}\right), $$

where *k* [m^2^_pore_] is the soil water permeability (Scheidegger 1957), *μ* [Pa s] is the dynamic viscosity of water, *ρ* [kg_water_ m^−3^_water_] is the density of water, $\overset{\sim }{p}$ [Pa] is the negative water potential in the soil, *g* [m s^−2^] is the gravitational acceleration, and $\hat{\boldsymbol{k}}$ [−] is the unit vector in the vertical downward direction. Water is assumed to be incompressible, which based on its bulk modulus and the conditions tested here, is valid as less than 0.01% volume change could occur.

We adopt the Van Genuchten formulation of the soil water retention characteristic relationship between the degree of soil saturation and the matric potential (Roose and Fowler 2004b; Genuchten and Th 1980). The soil water characteristic curve is denoted by the following function:

3 $$ f(S)=-{\left({S}^{-\frac{1}{m}}-1\right)}^{1-m}, $$

where *m*[−] is an empirically determined parameter associated with the shape of the soil water retention curve. Matric potential is calculated by the following equation:

4 $$ \overset{\sim }{p}=\tilde{p}_{c}f(S), $$

where $\tilde{p}_{c}\left(=\frac{\rho g}{\alpha}\right)$ [Pa] is the empirically determined soil characteristic potential. To quantify water permeability as a function of soil moisture, we again adopt the Van Genuchten formulation of the relative hydraulic conductivity based on the empirical soil water retention relationship (Genuchten and Th 1980; Roose and Fowler 2004b):

5 $$ K(S)={S}^{\frac{1}{2}}{\left(1-{\left(1-{S}^{\frac{1}{m}}\right)}^m\right)}^2. $$

Considering unsaturated soil conditions, we define the permeability as:

6 $$ k={k}_sK(S), $$

where *k*_*s*_[m^2^_pore_] is the soil water permeability under saturated conditions. As we wish to represent Eq. (1) in terms of saturation, we use a chain rule to convert the pressure gradient into a saturation gradient:

7 $$ \overset{\sim }{\mathbf{\nabla}}\overset{\sim }{p}=-\tilde{p}_{c}{f}^{\prime (S)}\ \overset{\sim }{\mathbf{\nabla}}S. $$

Factoring out the constant coefficients in front of the saturation gradient in Eq. (1), we obtain the soil water diffusivity constant as:

8 $$ {D}_0=\frac{\tilde{p}_{c}{k}_s}{\mu}\left(\frac{1-m}{m}\right), $$

where *μ* [Pa s] is the dynamic viscosity of water. The unsaturated diffusivity water relationship as:

9 $$ D(S)={S}^{\left(-\frac{1}{m}-\frac{1}{2}\right)}{\left({S}^{-\frac{1}{m}}-1\right)}^{-m}K(S). $$

Similarly, the expression for saturated hydraulic conductivity is given as:

10 $$ {K}_s=\frac{\rho g{k}_s}{\mu }. $$

Considering precipitation/irrigation conditions at the top of the domain, zero flux at the bottom, and substituting (10),(9),(8), and (5) into (2) and then (2) into (1), we obtain (Heppell et al. 2014; Roose and Fowler 2004b):

11 $$ \Big\{{\displaystyle \begin{array}{c}\phi \frac{\partial S}{\partial \overset{\sim }{t}}=\overset{\sim }{\nabla}\cdot \left({D}_0D(S)\overset{\sim }{\nabla }S-{K}_sK(S)\hat{\boldsymbol{k}}\right)-{\overset{\sim }{F}}_w,\kern0.5em \overset{\sim }{z}\in {\Omega}_{\mathrm{s}},\\ {}\hat{\boldsymbol{n}}\cdot \left({D}_0D(S)\overset{\sim }{\nabla }S-{K}_sK(S)\hat{\boldsymbol{k}}\right)=-\overset{\sim }{w},\kern0.5em \overset{\sim }{z}=0,\\ {}\hat{\boldsymbol{n}}\cdot \left({D}_0D(S)\overset{\sim }{\nabla }S\right)=0,\kern0.5em \overset{\sim }{z}={L}_P,\\ {}S\left(0,\overset{\sim }{z}\right)={S}_0,\kern0.5em \overset{\sim }{t}=0,\end{array}}\operatorname{} $$

where $\overset{\sim }{w}$ [m3water m-2surface s-1] is the net precipitation flux on the soil surface, *L*_*P*_[m] is the soil depth, and *S*_0_ [−] is the initial saturation state of the soil. The boundary and initial conditions will be discussed in greater detail in the methods section. We note that the saturation form Richards’ equation is valid only for unsaturated homogeneous soil, which applied to the simulation conditions modelled in this paper. Extending the model to either very dry or flooded soils would be possible by using the more complex potential form Richards’ equation (Duncan et al. 2018).

### Modelling root water uptake during growth

The uptake of soil water and its transport to the shoot is a fundamental function of the root system. In the rhizosphere, water moves into the root cortical tissue passively along the apoplastic and actively through the symplastic pathways (Steudle and Peterson 1998). As previous studies found that water movement through the root system is primarily passive, we focus on water movement across the apoplastic pathway. Passive flow is the result of pressure gradients between the soil and plant xylem; Fig. 1(d). Xylem vessels are tube like vessels formed of non-living cells (Frensch and Steudle 1989; Roose and Fowler 2004b). The volumetric flow rate through the xylem is denoted by (Frensch and Steudle 1989):

12 $$ {Q}_z=-{k}_z\left(\frac{\partial {\tilde{p}}_r}{\partial \tilde{z}}-\rho g\right), $$

where ${\tilde{p}}_r$ [Pa] is the root water pressure in the xylem tubes, $\tilde{z}$ [m] is the depth along the root, and *k*_*z*_ [m^4^ Pa^−1^ s^−1^] is the root axial conductivity, defined as (Frensch and Steudle 1989):

13 $$ {k}_z=\pi \frac{n_E{\left(\frac{d_E}{2}\right)}^4+{n}_L{\left(\frac{d_L}{2}\right)}^4}{8\mu }, $$

where *d*_*E*_ and *d*_*L*_ [m] are the early and late xylem vessel diameters respectively, and *n*_*E*_ and *n*_*L*_[−] are the number of open xylem vessels with respective mean diameters *d*_*E*_ and *d*_*L*_. In root systems the xylem diameters vary, but the assumed constant diameter simplifies the model and is sufficient to describe bulk transport (Frensch and Steudle 1989; Roose and Fowler 2004b) . Assuming that the root water uptake is dominated by the pressure difference between soil water and xylem water, we obtain the following expression for the volumetric water flux into the plant root:

14 $$ {q}_r={k}_r\left(\overset{\sim }{p}-\tilde{p}_{r}\right), $$

where *k*_*r*_ [m Pa^−1^ s^−1^] is the cortical (radial) water conductivity. For any axial partition down the length of the xylem, the mass balance requires that differences in volumetric flow along the xylem must be compensated by water moving radially between the plant root and the soil *i*. *e*. Δ*Q*_*z*_ = 2*πa q*_*r*_; Fig. 1(d). Therefore the expression describing the pressure balance between the root and the soil is given by (Frensch and Steudle 1989; Roose and Fowler 2004b)

15 $$ \left\{\begin{array}{c}{k}_z\frac{\partial^2{\tilde{p}}_r}{\partial {\tilde{z}}^2}=-2{\pi ak}_r\left(\tilde{p}-{\tilde{p}}_r\right),\kern0.5em \tilde{z}\in {\Omega}_r,\\ {}\frac{\partial {\tilde{p}}_r}{\partial \tilde{z}}-\rho g=0,\kern0.5em \tilde{z}={l}_0(t),\\ {}{\tilde{p}}_r={P}_{root},\kern0.5em \tilde{z}=0,\end{array}\right. $$

where *a*[m] is the nominal root radius, *l*_0_(*t*) [m] is the length of the primary (zero order) plant root at time *t*, *P*_*root*_ [Pa] is the driving pressure at the shoot (within it at the base), and the root domain Ω_r_(=[0,  *l*_0_(*t*)]) is growing in the soil domain (Fig. 1(c)). This model considers that plant roots grow in the soil, thus the domain is not static. However, as the focus is not on the root growth itself, a simplified model is implemented based on the work of Roose and Fowler (2004b). For a root of order *i*, the growth rate is defined as:

16 $$ \frac{\partial {l}_i}{\partial t}={r}_i\left(1-{l}_i/{l}_{i,f}\right),\kern0.5em t>0, $$

where *l*_*i*_ [m] is the immediate length of an *i*th order root, *r*_*i*_ [m s^−1^] is the growth rate of the *i*th order root, and *l*_*i*, *f*_[m] is the assumed maximum length of the *i*th order root. Following Roose and Fowler (2004b), the analytic expression used in this study is:

17 $$ {l}_i={l}_{i,f}+\left({l}_{i,0}-{l}_{i,f}\right){e}^{-\frac{r_it}{l_{i,f}}},t>0, $$

where *l*_*i*, 0_ [m] is assumed to be the initial root length for the initial condition. We assume that the primary root grows vertically into the soil.

### Considering first order lateral roots for water uptake

Following field evidence suggesting that primary and first order lateral roots contribute to the root water uptake processes (Varney et al. 1991), we consider a simplified model for lateral branching (Roose and Fowler 2004b). Considering a minimum non-branching length *l*_0, *a*_(=0.05[*m*]), we assume that first order lateral roots begin to branch from the primary root when *l*_0_(*t*) > *l*_0, *a*_ in the zone *z* ∈ [*l*_0, *a*_, *l*_0_(*t*) − *l*_0, *a*_) (Fig. 1e). As lateral root length enhances the radial water movement through the roots (Landsberg and Fowkes 1978), we approximate the contribution of the volumetric water flow rate in lateral roots as (Roose and Fowler 2004b):

18 $$ {Q}_{r_1}\approx \sqrt{2{\pi ak}_r{k}_{z,1}}\left(\overset{\sim }{p}\left(\hat{z}\right)-\tilde{p}_{r}\left(\hat{z}\right)\right), $$

where *k*_*z*1_[m^4^ Pa^−1^ s^−1^] is the xylem conductivity of the first order lateral roots, and $\hat{z}$ [m] is the location of a branch point on the primary root (Fig. 1e). Considering a uniform distribution of first order branching roots in the branching zone, the pressure balance in Eq. (15) becomes:

19 $$ \left\{\begin{array}{c}{k}_z\frac{\partial^2{\tilde{p}}_r}{\partial {\tilde{z}}^2}=-\left(2{\pi ak}_r+\sqrt{2{\pi ak}_r{k}_{z,1}\ }{\psi}_1(z)\right)\left(\tilde{p}-{\tilde{p}}_r\right),\kern1em \tilde{z}\in {\Omega}_r,\\ {}\frac{\partial {\tilde{p}}_r}{\partial \tilde{z}}-\rho g=0,\kern0.5em \tilde{z}={l}_0(t),\\ {}{\tilde{p}}_r=P,\kern0.5em \tilde{z}=0,\end{array}\right. $$

where *ψ*_1_(*z*) [m^−1^] is the first order lateral branching distribution per unit length of the main root, defined as:

20 $$ {\psi}_1(z)=\Big\{{\displaystyle \begin{array}{c}\Big\{1/{l}_{1,n},\kern0.5em \\ {}0,\end{array}}\kern1.25em {\displaystyle \begin{array}{c}{l}_{0,a}\le z\le {l}_0(t)-{l}_{0,a},\\ {} Otherwise,\end{array}}\operatorname{} $$

where *l*_1, *n*_ [m] is the intermodal distance between two side branches on the main root, i.e. 1/*l*_1, *n*_ is the number of first order root side branch points per main order root. As *ψ*_1_(*z*) represents the number of side branch points per unit length of the main root, it is number per unit length and the quantity hence has units 1/m. Solving for the root pressure, we obtain the expression for the sink term in the Richards’ equation (Roose and Fowler 2004b):

21 $$ {\overset{\sim }{F}}_w=\frac{2\pi a{k}_r+\sqrt{2\pi a{k}_r{k}_{z,1}}{\psi}_1(z)}{\pi {\left(a+{l}_{1,f}\cos \left(\beta \right)\right)}^2}\left(\overset{\sim }{p}(S)-\tilde{p}_{r}\right), $$

where *l*_1, *f*_[m] is the maximum length of the first order branching root, and *β*[rad] is the branching angle (assumed *π*/3). The total volumetric water uptake rate is estimated as:

22 $$ {\tilde{Q}}_{r,w}=\pi {\left(a+{l}_{1,f}\cos \left(\beta \right)\right)}^2{\int}_0^{l_0}{\tilde{F}}_wd\tilde{z}. $$

For assessing the transpiration fluxes over the soil surface, we consider a root flux over a cubic meter of land area:

23 $$ {\overset{\sim }{J}}_{r,w}=\frac{{\overset{\sim }{Q}}_{r,w}}{{\overset{\sim }{A}}_{ss}}, $$

where ${\tilde{A}}_{ss}$ is the soil surface area, which we consider 1 m^2^_soil_. We point out that the lateral roots extend the zone of influence of the uptake sink term. Details regarding their depth influence and their role in solute uptake will be further described in the latter sections.

### Coupling nutrient transport and flow through partially saturated soil

To consider the impact that root hairs have at the field scale, we employed the nutrient conservation equations in soil (Nye and Tinker 1977; Roose and Fowler 2004a; Roose et al. 2001; Tinker and Nye 2000), given by:

24 $$ \frac{\partial }{\partial \overset{\sim }{t}}\left(\left(b+\phi S\right)\overset{\sim }{c}\right)+\overset{\sim }{\nabla}\cdot \left(\overset{\sim }{c}\overset{\sim }{\boldsymbol{u}}\right)=\overset{\sim }{\nabla}\cdot \left({D}_f{\left(\phi S\right)}^{d+1}\overset{\sim }{\nabla}\overset{\sim }{c}\right)-\overset{\sim }{F},\kern0.5em \overset{\sim }{z}\in {\Omega}_{\mathrm{s}}, $$

where $\tilde{c}$ [mol_solution_ m^−3^_water_] is the nutrient concentration in the soil water phase, $\tilde{F}$ [mol_solution_ m^−3^_bulk_ s^−1^] is the volumetric nutrient uptake rate by the plant roots (${\tilde{F}}_0$ by primary roots and ${\tilde{F}}_1$ by first order laterals, (Fig. 1(c)), $\tilde{\boldsymbol{u}}$ [m^3^_water_ m^−2^_bulk_ s^−1^] is the volumetric water flux that advects mobile nutrients (Fig. 1(a)), *D*_*f*_ [m^2^ s^−1^] is the solute diffusion in the water phase (Fig. 1(a)), *d*[−] is a soil tortuosity factor, and *b*[mol_ads_ mol^−1^_des_ m^3^_water_ m^−3^_bulk_] is the soil buffer power (i.e. the ratio between the nutrient particles adsorbed and desorbed to the soil particle surfaces, Fig. 1(b)). We note that as tortuosity, soil moisture, and advection are explicitly considered in our modelling scheme, the effects of dispersion should be sufficiently accounted for.

For this study, the nutrient of interest is P. Phosphorus applied in the surface layer as fertiliser may move down the soil profile and is depleted by root nutrient uptake. Considering the boundary conditions, and substituting (10),(9),(8) and (5) into (2) and then (2) into (24) yields:

25 $$ \left\{\begin{array}{c}\frac{\partial }{\partial \tilde{t}}\left(\left(b+\phi S\right)\tilde{c}\right)+\tilde{\mathbf{\nabla}}\cdotp \left(-\left({D}_0D(S)\tilde{\mathbf{\nabla}}S-{K}_sK(S)\hat{\boldsymbol{k}}\right)\tilde{c}\right)=\tilde{\mathbf{\nabla}}\cdotp \left({D}_f{\left(\phi S\right)}^{d+1}\tilde{\mathbf{\nabla}}\tilde{c}\right)-\tilde{F},\kern0.5em \tilde{z}\in {\Omega}_{\mathrm{s}},\\ {}\hat{\boldsymbol{n}}\cdotp \left({D}_f{\left(\phi S\right)}^{d+1}\tilde{\mathbf{\nabla}}\tilde{c}+\left({D}_0D(S)\tilde{\mathbf{\nabla}}S-{K}_sK(S)\right)\tilde{c}\right)=\tilde{\varrho},\kern0.5em \tilde{z}=0,\\ {}\hat{\boldsymbol{n}}\cdotp \left({D}_f{\left(\phi S\right)}^{d+1}\tilde{\mathbf{\nabla}}\tilde{c}+\left({D}_0D(S)\tilde{\mathbf{\nabla}}S-{K}_sK(S)\right)\tilde{c}\ \right)=0,\kern0.5em \tilde{z}={L}_P,\\ {}\tilde{c}\left(0,z\right)={\tilde{c}}_0\left(\tilde{z}\right),\kern0.5em \tilde{t}=0,\end{array}\right. $$

where the initial condition ${\tilde{c}}_0\left(\tilde{z}\right)$ [mol_solution_ m^−3^_water_] represents an initial distribution of P along the depth, and $\tilde{\varrho}$ [mol m^−2^ s^−1^] is the rate of fertilizer application. The results are represented as $\left(b+\phi S\right)\tilde{c}$ [mol_P_ m^−3^_bulk_], which consider the total concentration of both adsorbed and dissolved P in the bulk soil volume. The boundary condition at the bottom of the domain (no flux) is a simplification based on the mobility of P in soil. As we’re considering transport at the field scale (*L*_*p*_ = 1 *m*), transport of P via diffusion (solving $x\approx \sqrt{t{D}_f}$) results in P taking 40 years to move 0.5 m. As the buffer power is so high (up to 239(Barber 1995)), P readily binds to soil, leaving it mostly immobile, thus we expect the uptake by roots to be localized.

### Modelling P uptake during growth

Phosphorus uptake by roots occurs in two different steps. The first occurs as the primary root is growing through the system, and subsequently, the lateral roots begin to grow and take up nutrients. For this purpose, the P uptake sink in the solute transport equation is split into two terms

26 $$ \tilde{F}={\overset{\sim }{F}}_0+{\overset{\sim }{F}}_1, $$

where ${\tilde{F}}_0$ and ${\tilde{F}}_1$ are the dimensionless volumetric nutrient uptake rates by the primary and lateral roots, respectively. For the primary roots, the uptake rate derived from the matched asymptotic solution of the radial uptake flux (Roose et al. 2001) is defined by (Roose and Fowler 2004a):

27 $$ {\tilde{F}}_0=\frac{2{\Lambda}_0\tilde{c}}{1+\tilde{c}+{L}_0\left(\tilde{z},\tilde{t}\right)+\sqrt{4\tilde{c}+{\left(1-\tilde{c}+{L}_0\left(\tilde{z},\tilde{t}\right)\right)}^2}}, $$

with Λ_0_ is defined as:

28 $$ {\Lambda}_0=\frac{2a{F}_m}{{\left(a+{l}_{1,f}\cos \left(\beta \right)\right)}^2{K}_m}, $$

where *F*_*m*_(=3.26×10^−8^ mol m^−2^ s^−1^) is the maximum rate of root nutrient uptake for P, and *L*_0_ is defined as:

29 $$ {L}_0\left(\tilde{z},\tilde{t}\right)=\frac{\lambda_0}{2\ {S}^{d+1}}\ln \left(\left({\alpha}_0\tilde{t}+{\alpha}_{00}\mathit{\ln}\left(1-\left(\frac{\tilde{z}}{K_0}\right)\right)\ \right)\left(\frac{S^{d+1}}{1+\delta S}\right)+1\right). $$

In this equation *δ* = *ϕ*/*b*, *λ*_0_ = *F*_*m*_*a*/(*D*_*f*_*ϕ*^*d* + 1^*K*_*m*_), ${\alpha}_0=4{e}^{-\gamma}\left(\frac{D_f{\phi}^{d+1}}{a^2b}\right)$, *γ*≈ 0.5772 is the Euler-Mascheroni constant (Lagarias 2013; Roose and Fowler 2004a), and ${\alpha}_{00}=4{e}^{-\gamma}\left(\frac{D_f{\phi}^{d+1}}{a^2b}\right)\left(\frac{l_{0,f}}{r_0}\right)$, where *r*_0_ [m s^−1^] is the maximum primary root growth rate. For the uptake by the first lateral roots, the model considers the summation of the uptake by all of the lateral roots in the branching zone:

30 $$ {\tilde{F}}_1={\int}_{\hat{\tilde{z}}}^{\tilde{z}}\frac{2{\Lambda}_1\tilde{c}\ {\psi}_1\left(z^{\prime}\right) dz^{\prime }}{1+\tilde{c}+{L}_1\left(\tilde{z},\tilde{t};z^{\prime}\right)+\sqrt{4\tilde{c}+{\left(1-\tilde{c}+{L}_1\left(\tilde{z},\tilde{t};z\prime \right)\right)}^2}}, $$

where $\hat{\tilde{z}}$[m] is the location of a nodal branching point, and:

31 $$ {\Lambda}_1=\frac{2a{F}_m}{\cos \left(\beta \right){\left(a+{l}_{1,f}\cos \left(\beta \right)\right)}^2{K}_m}, $$

where *l*_*n*, 0_[m] is the nodal distance of lateral roots from one another. Similar to *L*_0_, *L*_1_ is defined as:

32 $$ {L}_1\left(\tilde{z},\tilde{t};z^{\prime}\right)=\frac{\uplambda_1}{2{S}^{d+1}}\ln \left(\left({\alpha}_1\tilde{t}+{\alpha}_{11}\ln \left(\left(1-\left(z^{\prime }+{l}_a0\right)\right)\right)+{\alpha}_{111}\ln \left(\left(1-\frac{\tilde{z}-z^{\prime }}{l_{1,f}\cos \left(\beta \right)}\right)\right)\right)\left(\frac{S^{d+1}}{1+\delta S}\right)+1\right), $$

where *λ*_1_ = *F*_*m*_*a*_1_/(*D*_*f*_*ϕ*^*d* + 1^*K*_*m*_), where *a*_1_[m] is the radius of the first order roots, ${\alpha}_1=4{e}^{-\gamma}\left(\frac{D_f{\phi}^{d+1}}{a_1^2b}\right)$, ${\alpha}_{11}=4{e}^{-\gamma}\left(\frac{D_f{\phi}^{d+1}}{a_1^2b}\right)\left(\frac{l_{0,f}}{r_0}\right)$, and ${\alpha}_{111}=4{e}^{-\gamma}\left(\frac{D_f{\phi}^{d+1}}{a_1^2b}\right)\left(\frac{l_{1,f}}{r_1}\right)$ where *r*_1_ [m s^−1^] is the maximum lateral root growth rate (Roose and Fowler 2004a).

**Fig. 2:**
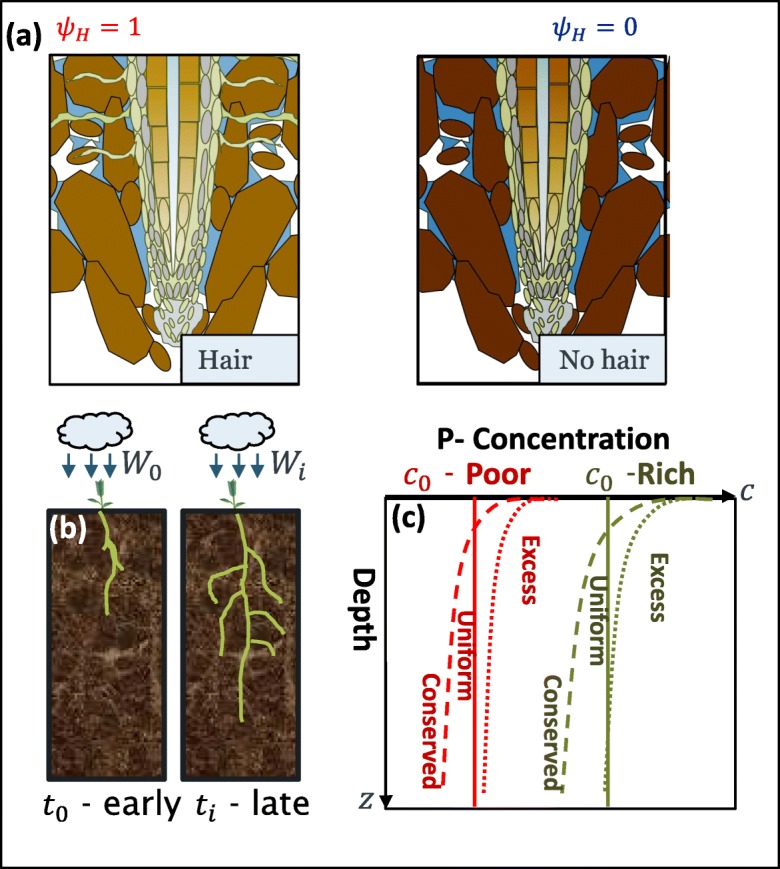
Full illustration of the parametric study. **a** Simulations are run with and without root hairs. Precipitation conditions **b** are prescribed as instantaneous (*t*_0_) or midway through the simulation (*t*_*i*_*).***c** Simulations are run considering rich and poor initial P concentrations, and uniform and decaying distributions along the depth. Two decaying distributions are considered. The P decaying profile labelled conserved has an equivalent initial volume as that of the uniform P profile. The decaying profile labelled excess decays to the uniform distribution. The results consist of 24 simulations in total

Importantly, the solute uptake by lateral roots (Eq. (30)) requires knowledge about the zone of influence of the given root $\left(\hat{\tilde{z}},\tilde{z}\right]$ (Fig. 2). For any given branching point $\hat{\tilde{z}}$, the lateral root that branches from the primary root has a zone of influence at a range of given depths (Fig. 2) defined as:

33 $$ \tilde{z}=\hat{\tilde{z}}+{l}_{1,f}\cos \left(\beta \right)\left(1-{e}^{-\frac{r_1\tilde{t}}{l_{1,f}}}\left(1-{\left(\hat{\tilde{z}}+\frac{l_{a,0}}{l_{0,f}}\right)}^{-\frac{r_1{l}_{0,f}}{r_0{l}_{1,f}}\tilde{t}}\right)\right), $$

thus for any $\tilde{z}$ [m] at any point in time, $\hat{z}$ [m] has to be numerically computed. Similar to the water uptake rate, the total P uptake rate is estimated as:

34 $$ {\overset{\cdotp }{\tilde{m}}}_{r,P}=\pi {\left(a+{l}_{1,f}\cos \left(\beta \right)\right)}^2{\int}_0^{l_0}\tilde{F}d\tilde{z}, $$

and the mass of P taken up by the plant roots is:

35 $$ {\tilde{m}}_{r,P}={\int}_0^t{\overset{\cdotp }{\tilde{m}}}_{r,P}\left(\tau \right) d\tau \kern0.5em , $$

where *τ* [s] is a dummy variable for integration. All of the above equations were non-dimensionalised and simplified for the subsequent implementation. For details, see Supplementary Material S2. We make note that the uptake sink term does not consider advection through the roots. This simplification was made on the basis that the contribution of advection in the roots only account for 1-2% of the total uptake (Roose and Kirk 2009). Furthermore, P uptake in UK soils is diffusion driven, as the Péclet number *Pe* < 10^−3^ (Roose and Kirk 2009).

### Assessing the impact of root hairs

While there is uncertainty regarding the impact that root hairs have on water uptake (Carminati et al. 2017), we make a deliberate choice to neglect root hair water uptake and only account for hair P uptake. Root hairs are single cell extensions out of single root epidermal cells. Thus they do not contain the vasculature (i.e. xylem, phloem etc) that plant roots have (Kozinka and Kolek 1992; Lambers and Colmer 2005). Therefore, transport of water through the root hairs is at best reliant on osmotic gradients and diffusive transport through the cells apoplastic pathways. Furthermore, root hairs are effectively under high tugor pressure, thus the pressure inside the hairs is on the order of 500-1000 kPa, which is often greater than in the soil pore space (Lew 1996). Thus, the rates of water movement in the apoplastic pathways will be slower (if not zero) than water movement through symplastic pathways, which are driven by pressure gradients in the xylem. Thus, we neglect the impact that root hairs may have towards water uptake. We note that root hairs may provide indirect advantages to plant water acquisition by means of physical root hair configurations in the pore space (i.e. access to finer pore spaces retaining water, physically altering liquid bridges, etc.), however, future studies are required to elucidate these intricacies (Carminati et al. 2017).

The model considers a semi-empirical expression accounting for P uptake by root hairs. We assumed that root hairs were uniformly distributed along the root surface denoted as *ψ*_*H*_. We consider that the root uptake rate of P expands to:

36 $$ \tilde{F}=\frac{1}{2}\left({\psi}_H+1\right){\tilde{F}}_r, $$

where *ψ*_*H*_ is the root hair effect along the roots, and ${\tilde{F}}_r$ is the uptake contribution of roots with no root hairs. Simulations were run considering *ψ*_*H*_ = 1, representing wild type roots with hairs and *ψ*_*H*_ = 0, modelling hairless mutants (Gahoonia et al. 2001; Suzuki et al. 2003); Fig. 2(a).

### Comparing the impact of precipitation initiation

Precipitation is simulated using the soil surface net water flux boundary condition in Eq. (11). We model two precipitation scenarios to determine the influence that:

37 $$ w=\Big\{{\displaystyle \begin{array}{c}0,\kern0.5em t<{t}_{rain},\\ {}W,\kern0.5em t\ge {t}_{rain},\end{array}}\operatorname{} $$

where *W* = 0.05 [−] is the dimensionless net water flux in the soil surface and ${\tilde{t}}_{rain}$ is the dimensionless initiation time for precipitation ($t=\tilde{t}\left({D}_0/\left(b{l}_{0,f}^2\right)\right)$, see SI 2 for more details). The two initiation times selected are for the initiation of the simulation (*t*_*rain*_ = 0) and midway through the simulation (precipitation is modelled as a smoothed step function that attains its maximum at *t*_*rain*_ = *t*_*final*_/2); Fig. 2(b). The dimensional *t*_*final*_ was 150 days for the simulation, as this was sufficient for the inner seasonal dynamics to stabilize. Long term simulations considering multiple growth seasons was also considered and will be described in later sections.

### Initial P concentration and distribution

We assess the effects that different initial P distributions have on changes to P concentrations in soil and total P uptake. We consider six separate P distributions scenarios (Fig. 2(d)). The first two scenarios are initialized with constant P concentration along the depth, one at a high concentration and one at low (simulations are denoted as uniform). The subsequent two scenarios consider P concentrations exponentially decaying along the depth converging to the concentration value of the constant simulations (denoted as excess). The final two scenarios simulate an initially decaying concentration of P along the depth similar to the previous, however, the integrated volume of P along the active root depth is equivalent to the uniform scenarios (denoted as uniform). Thus, the initial P concentration in the soil is defined as:

38 $$ {c}_0^{\ast}\left(z;\zeta \right)={c}_{0,c}^{\ast }{e}^{-\zeta z}, $$

where *ζ* = 0 or *ζ* = 1 depending on whether the model is considering a constant or decaying distribution of P along the depth ($c=\tilde{c}/{K}_m$, see SI 2 for more details on non-dimensionalisation). Up to the maximum rooting depth (*K*_0_), we assume that the cumulative volume of P for both the uniform distribution and decaying distribution is the same (${\int}_0^{z_f}{c}_0^{\ast}\left(z;0\right) dz={\int}_0^{z_f}{c}_0^{\ast}\left(z;1\right) dz$), thus the relationship between the coefficient is:

39 $$ {c}_0^{\ast}\left(z;1\right)=\left(\frac{z_f}{1-{e}^{-{z}_f}}\right){c}_0^{\ast}\left(z;0\right), $$

where ${c}_{0,d}^{\ast }$ is the coefficient for the decaying initial distribution, and ${\tilde{c}}_{0,c}^{\ast }$ is the coefficient or magnitude of the uniform distribution along the depth. A general expression that would consider any initial concentration distribution for both the uniform and conserved scenarios is expressed as:

40 $$ {c}_0(z)={\left(\frac{z_f}{1-{e}^{-{z}_f}}\right)}^{\zeta }{c}_{0,c}^{\ast }{e}^{-\zeta \tilde{z}}, $$

where we consider *z*_*f*_ = 1 [−], and ${c}_{0,c}^{\ast }$ [−] is the initial magnitude of the concentration magnitude (chosen to be ${c}_{0,c}^{\ast }=0.1$ and ${c}_{0,c}^{\ast }=0.33$, Fig. 2(d)). To include the excess scenario, we modify the expression:

41 $$ {c}_0(z)={\left(\chi +{\left(\frac{z_f}{1-{e}^{-{z}_f}}\right)}^{1-\chi}\right)}^{\zeta }{c}_{0,c}^{\ast }{e}^{-\zeta z}, $$

where *χ* is the excess coefficient (either 0 or 1). Thus, when *χ*= 0, Eq. (41) becomes Eq. (40). Considering all of the combinations of the simulations (including with and without root hairs, immediate and subsequent rainfall, high and low P concentrations, constant and decaying distributions, and excess volume vs conserved volume), the model ran for 24 unique realizations (Fig. 2). The full sweep of simulated studies is noted in Table 2.

**Table 2:** List of parametric cases examined in the study. Due to overlap in certain combinations, the final number of unique case studies carried out in this simulation is 24

	*t*_*rain*_	${c}_{0,c}^{\ast }$	*ζ*	*ξ*	*ψ*_*H*_
1	0	0.1	0	0	0
2	0.025	0.33	1	1	1

### Assessing long term effects after multiple cropping seasons considering real rain data

**Fig. 3:**
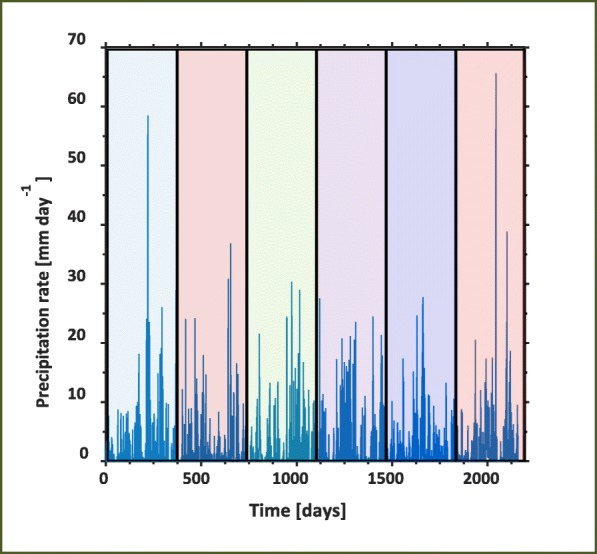
Precipitation data used for 6 years-long simulations. Rainfall data were taken from Invergowrie, Scotland from the beginning of 2004 to the end of 2009

We conduct a final set of simulations to better understand the impact of root hairs on soil P over several growing seasons. The model considers a rich uniform distribution of P along the soil depth. The model is run over a 6 years timespan (2004-2010) using precipitation data provided by the Scottish Crop Research Institute (currently part of The James Hutton Institute, Dundee, Fig. 3).

Daily precipitation rates were used as model inputs for the flux. For all 6 years, roots are simulated to grow in the soil for the first 150 days of the year (starting on January 1st, 2004) and are removed for the remaining 215 days. Due to the long durations of the simulations and the sporadic rainfall events, the soil moisture is susceptible to reaching saturation, which could cause numerical instabilities. In order to mitigate this issue, we scaled down the inward fluxes as soil water contents approached saturation. This was implemented by effectively changing Eq. (11) to:

42 $$ \left\{\begin{array}{c}\left(1-{H}_S(S)\right)\phi \frac{\partial S}{\partial \tilde{t}}=\tilde{\nabla}\cdot \left({D}_0D(S)\tilde{\nabla}S-{K}_sK(S)\hat{k}\right)-{\tilde{F}}_w,\tilde{z}\in {\varOmega}_{\mathrm{s}},\\ {}\hat{n}\cdot \left({D}_0D(S)\tilde{\nabla}S-{K}_sK(S)\hat{k}\right)=-\left(1-{H}_S(S)\right)\tilde{w},\tilde{z}=0,\\ {}\hat{n}\cdot \left({D}_0D(S)\tilde{\nabla}S\right)=0,\tilde{z}={L}_P,\\ {}S\left(0,\tilde{z}\right)={S}_0,\tilde{t}=0,\end{array}\right. $$

where *H*_*S*_(*S*) is a smoothed Heaviside step function that turns off the flux as the domain approaches saturation (Duncan et al. 2018).

### Soil properties

Simulations conducted in the study consider soil conditions studied as part of a field experiment at the James Hutton Institute that considers hairy and hairless barley roots. The soil properties used in the study were quantified from Bullionfield in Dundee (56^∘^27′ 39′′ N, 3^∘^04′11′′ W) (Naveed et al. 2017). The soil is a Dystric Cambisol with a sandy loam texture consisting of 16% clay, 34% silt, and 60% sand. It had a volumetric water content of 0.32 [m^3^_water_ m^−3^_bulk_] at zero tension and a residual volumetric water content of 0.14 [m^3^ m^−3^] (Liang et al. 2017). The full list of soil physical properties could be found in Table 1.

### Model validation

Though there are limited data pertaining to the conditions that this current study is modelling, a qualitative model validation was conducted based on general soil profile characteristics. We highlight a comparison between soil saturation depth profiles over time predicted by our model and measured under drought conditions considering root water uptake (Volaire and Thomas 1995). Similarly, we compare trends in the soil profile of data containing P in solution (*c*_*s*_ = *ϕSc* [mol_des_ m^−3^_bulk_]) to our model predictions based on the influence of growing plant roots(Gahoonia et al. 1994). As the measurements were conducted on different spatial scales and different P concentrations, we compare normalized concentrations between their measured data and our model output:

43 $$ \overline{c}=\frac{c_s-\min \left({c}_s\right)}{\max \left({c}_s\right)-\min \left({c}_s\right)}. $$

## Results

### Model validation

**Fig. 4:**
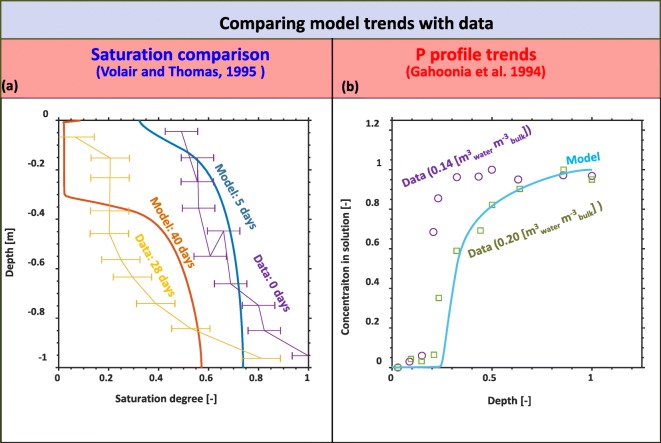
Qualitative model evaluation. **a** Comparing trends in saturation degree profile trends between the model output and measured field observations (Volaire and Thomas 1995) for two separate time periods (near the beginning of the experiment (days 0-5) and in the middle of the experiment (days 28-30). **b** Comparing the general trends between modelled and measured normalized P concentrations in solution (Gahoonia et al. 1994)

Model evaluation using limited data found in the literature is shown in Fig. 4. Modelled soil moisture profiles were run assuming no rain over the first 30 day period (Fig. 4(a)). Spatial and temporal trends for the saturation degree appear to qualitatively follow those seen in the experimental data (Fig. 4(a)), where the root uptake appears to influence the upper layer more dominantly than at the lower depths. We note that our simulations make use of a different soil and different root profiles, which would account for the differences seen in the model results and the field data. The P concentration in solution is illustrated in Fig. 4(b). Though the overall magnitudes and scale of the measurements and models are different, the general trends based on the influence of the plant roots are similar.

### Root-soil water dynamics under variable precipitation conditions

**Fig. 5:**
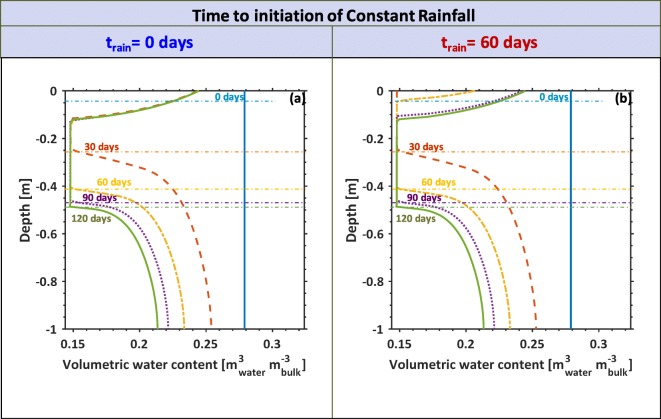
Soil volumetric water content [m^3^_water_ m^−3^_bulk_] profile considering root growth and water uptake for two precipitation scenarios. The scenario in (**a**) illustrates the water profile evolving over 120 days considering immediate and constant irrigation from the beginning of the simulation. Scenario (**b**) illustrates the soil water profile evolving considering precipitation initiating after 60 days. The 5 days illustrated in both (a) and (b) are for 0, 30, 60, 90, and 120 days coloured in blue, orange, yellow, purple, and green respectively. Maximum depth of the rooting zone for a given day is indicated by coloured dashed horizontal lines, where 0, 30, 60, 90, and 120 days correspond to the colours blue, orange, yellow, purple, and green respectively. Simulations consider residual water content as *θ*_*r*_ = 0.14 [m^3^ m^−3^] and a characteristic soil pressure $\tilde{p}_{c}=6.7\times {10}^3$ [Pa]

The resulting profiles for the two separate rainfall simulations are illustrated in Fig. 5. Using continuous precipitation initialized from *t*= 0 days (Fig. 5(a)), the simulations resulted in a gradual increase in the overall soil water content along the soil profile. After 60 days, water content had noticeably decreased near the bottom of the rooting zone. By 90 days, the soil water content near the root tip (˜ 0.5 m) flattened out near the residual water content, as the plant root suction can no longer exceed the soil matric potential. Different characteristic behaviour was observed in Fig. 5(b), where the initialization of the rain begins at *t*= 60 days and reached steady state at *t* = 75 days (simulated as a smooth step function where smoothing was made over the 15 day period). The profile initially dries from the soil surface down to the depth just behind the root tip location (0-30 days). On day 60, the soil surface begins to wet due to the precipitation. By days 90 and 120, the profiles are the same for precipitation at t = 0 or t = 60 days.

**Fig. 6:**
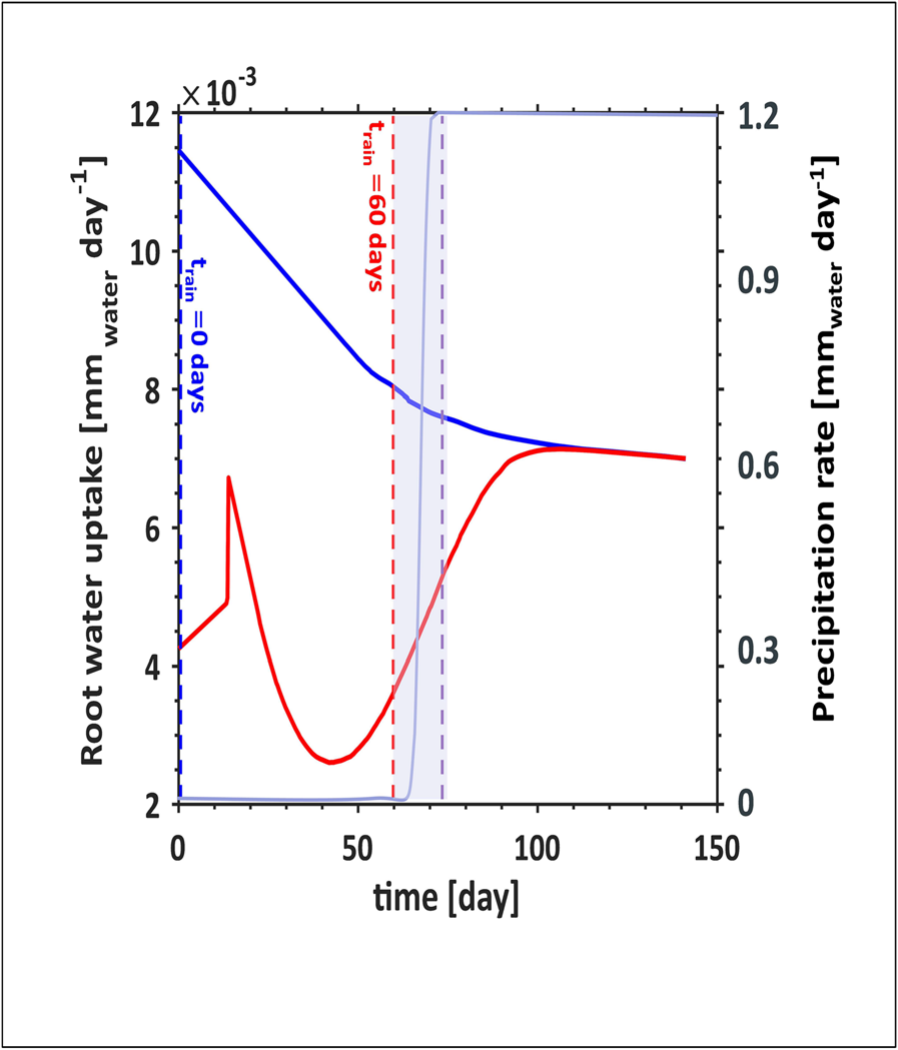
Total root water uptake rate as a function of time. Water uptake was integrated along the rooting depth and around the rooting zone (Eq. (22)) for two precipitation scenarios. The blue curve illustrates the root water uptake dynamics considering the scenario with immediate and constant irrigation from the beginning of the simulation. The red curve illustrates the root water uptake dynamics in response to zero irrigation for the first 60 days, increasing as a smoothed step to reach steady state by day 75 (plotted in the purple highlighted region with the axis on the right)

Despite the similarities in the soil moisture profiles, the root water uptake behaviour is very different for the two rainfall scenarios (Fig. 6). When the rain initialized at the beginning of the simulation (blue curve in Fig. 6), the root water uptake initiates at a maximal value of 11.5×10^−3^ mm day^−1^ and decays down. The root water uptake begins to stabilize after 100 days, tending towards 7×10^−3^ mm day^−1^. The growth scenario when precipitation is delayed for 60 days (red curve in Fig. 6) shows a rapid increase in water uptake increases following the initial simulation, but quickly drops off after 20 days. The water uptake rate remains low until precipitation begins (from 60 days), and soon reaches the uptake rate of the simulation conducted in the blue curve.

### Root solute uptake from uniform P distribution with depth

**Fig. 7:**
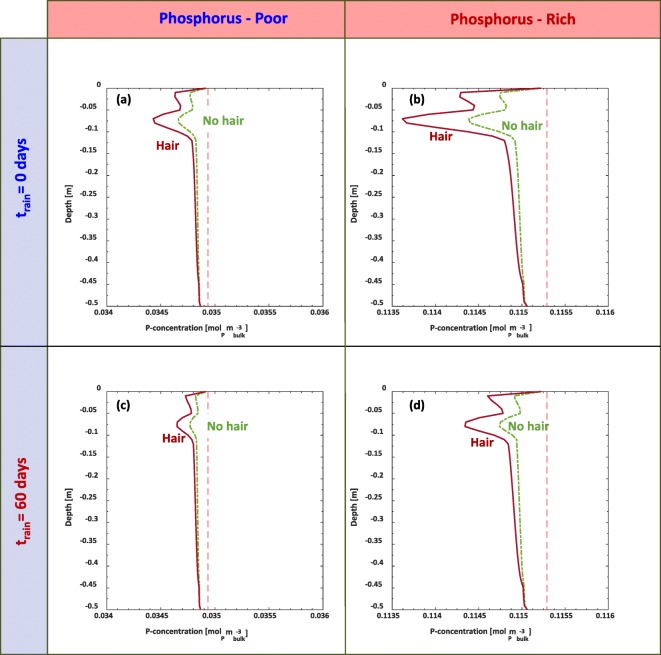
Soil phosphorus concentration [mol_P_ m^−3^_bulk_] profile considering root growth and P uptake for two precipitation scenarios, two initial uniform P distributions, and considering with (*ψ*_*H*_=1) and without (*ψ*_*H*_=0) root hairs. The scenario in (**a**) and (**b**) illustrates the final P profile after 150 days considering immediate and constant irrigation from the beginning of the simulation, while (**c**) and (**d**) consider precipitation initiating at day 60. The initial magnitude of the P concentration in (a) and (c) are at 0.2776 mol_P_ m^−3^_bulk_, while the initial magnitude of the P concentration in (b) and (d) are 0.916 mol_P_ m^−3^_bulk_. We note that the subdomain in the figures focusses on the rooting zone, not the full domain

Profiles of the total P concentrations based on an initial uniform distribution down the soil depth are plotted in Fig. 7. Root P uptake over the course of 150 days demonstrates small changes in the overall P concentrations. The overall P in the domain is less for the simulations influenced by root hairs. Reduction in P profiles appears more pronounced in the cases where rain is initiated at t_rain_ = 0 days (Fig. 7(a-b)) compared to the delayed rain scenarios (Fig. 7(c-d)). It is worth noting that the overall change in the P profiles are marginal for all simulations, resulting in a maximum percent change below 2% of the initial value.

### Root nutrient uptake from declining P distribution along the depth

**Fig. 8:**
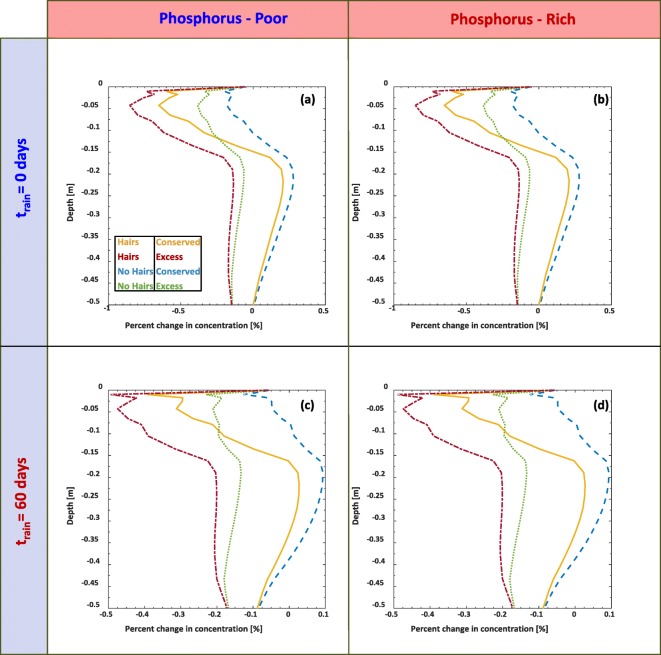
Profile of the percent difference of P with respect to the initial values at each depth considering root growth and P uptake for two precipitation scenarios, four initial decaying P distributions, and considering with (*ψ*_*H*_=1) and without (*ψ*_*H*_=0) root hairs. The scenario in (a) and (b) illustrates the final P profile after 150 days considering immediate and constant irrigation from the beginning of the simulation, while (c) and (d) consider precipitation initiating at t = 60 subsequent to the simulation. We note that the subdomain in the figures focusses on the rooting zone, not the full domain

Simulation results that have soil P concentration decreasing exponentially with depth were plotted as percent differences on Fig. 8 in order to provide a clearer comparison of the relative changes at each depth. Considering first the conserved volume simulations, the largest percent changes occurred in the t = 0 days rainfall (Fig. 8(a-b)), with over a 0.5% decrease in P at the maximum root depth due to the influence of root hairs. The remaining P in the hairless simulation was consistently greater in magnitude than their wild type counterpart simulations for both excess and conserved distributions in Fig. 8.

### Impact of water dynamics vs root hairs on total P uptake

**Fig. 9:**
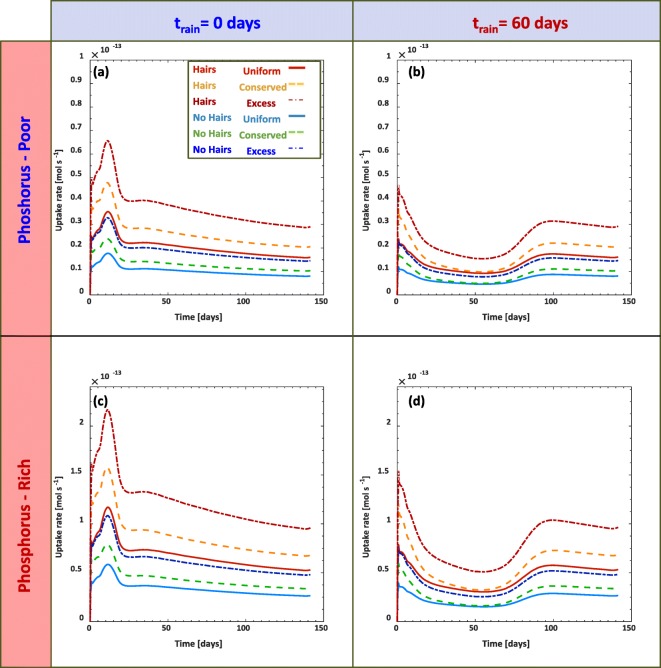
Root phosphorus uptake rate over time as impacted by each of scenarios. Each of the scenarios considers simulations with root hairs (*ψ*_*H*_=1) and without root hairs (*ψ*_*H*_ = 0) for uniform initial phosphorus distribution (solid curves), mass conserved decaying distribution (dashed), and excess mass decaying distribution (semi dashed). Plots in (a) and (b) consider simulations with initially low phosphate content in the soil, while (c) and (d) consider initially high phosphate content in the soil. Simulations in (a) and (c) consider rainfall initiated from the beginning of the simulation, while (b) and (d) consider precipitation after 60 days

The total P uptake rates are compared for all of the simulations in Fig. 9. Comparing first the low P vs high P scenario with precipitation throughout the simulations (Fig. 9(a) and (c)), shows that the uptake rates for all of the simulations was lower in the low P scenario than in the high P scenario, proportional to the volume of P in the system. Because the low P scenario has 70% less total soil P than the high P scenario, the reduction from the P uptake rate from the high P scenario (Fig. 9(c)) to the low P scenario (Fig. 9(a)) appears to also scale back by 70%. Uptake rates were, as expected, consistently greatest for the excess distributions, likely attributed to the scaling effect that the uptake rates have with the concentrations of P in the system.

For all of the scenarios, P that distributed uniformly had lower uptake rates than if P decreased exponentially with depth. This was likely due to a locally increased concentration of P as the root initially grows down into the soil domain. Simulations of the hairless roots consistently resulted in a 50% reduction in the maximum uptake rate in comparison to the hairy root counterpart simulations.

One of the most striking results was the overall change in dynamics when comparing the simulations with constant precipitation throughout the simulations (Fig. 9(a) and (c)) and the simulations with precipitation initiating after 60 days (Fig. 9(b) and (d)). Where the P uptake in Fig. 9(a) and (c) peaks at 15 days, with delayed rainfall simulations (Fig. 9(b) and (d)) uptake rates never achieve the same maximal values. The reduction in the P uptake rate ranged between 30 and 55% for the different scenarios.

**Fig. 10:**
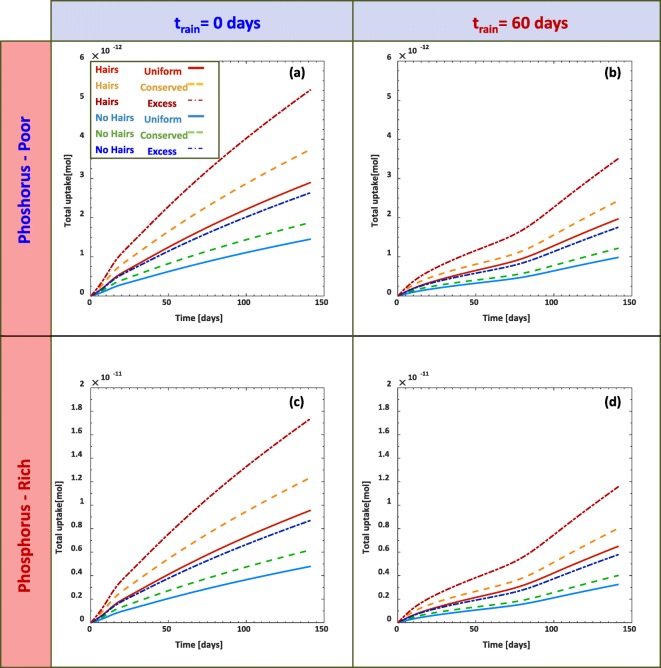
Total phosphorus uptake by plant roots over time as impacted by each of scenarios. Each scenario considers simulations with root hairs (*ψ*_*H*_=1) and without root hairs (*ψ*_*H*_ = 0) for uniform initial phosphorus distribution (solid curves), mass conserved decaying distribution (dashed), and excess mass decaying distribution (semi dashed). Plots in (a) and (b) consider simulations with initially low phosphate content in the soil, while (c) and (d) consider initially high phosphate content in the soil. Simulations in (a) and (c) consider rainfall initiated from the beginning of the simulation, while (b) and (d) consider precipitation after 60 days

The total quantities of P can also be observed by integrating the uptake rates over time (Fig. 10). Within the first 50 days, the root uptake doubles the quantity of P in the immediate rain scenario (Fig. 10 (a) and (c)) as compared to the delayed rain scenario (Fig. 10 (b) and (d)). After the rain initiates in the t_rain_ = 60 days scenario (Fig. 10 (b) and (d)), the uptake rate rapidly increases. Thus, the difference between in the quantities between the two rain scenarios range between a reduction of 33-35% the total P taken up by the plants.

### Impact of root hairs over several years

**Fig. 11:**
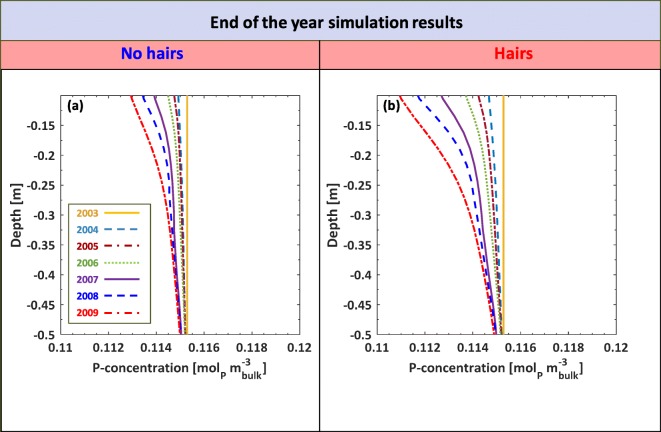
Soil P concentration after multiple growing seasons. (a) illustrates the relative impact that hairless mutants would have on the soil P compared to wild type hairy roots (b). Each curve depicts the end of the year P distribution along the soil depth assuming an initially uniform distribution at the beginning end of 2003 and simulating successive seasons up to the end of 2009. We note that the subdomain in the figures focusses on the rooting zone, not the full domain

A comparison of changing soil P concentration over time between roots with no hairs and with hairs for a 6 year period is plotted in Fig. 11. Roots are only present in the simulations during the growing season, so the end of year plots that are illustrated when roots were absent allow the system to equilibrate and smoothen. Over time the differences in P concentration with depth between roots with and without root hairs became more pronounced. The minimum concentration along the depth by the end of 2009 for the no hair scenario was about 0.113 [mol_p_ m^−3^_bulk_], whereas the minimum concentration was 0.111 [mol_p_ m^−3^_bulk_] for the simulations with hairs.

## Discussion

At the field scale, the modelling in this paper has demonstrated a large impact of the presence of root hairs and environmental conditions on P uptake by plants. We enhanced a model that considers root-soil interaction at the field scale by considering unsaturated soil moisture conditions and solute transport.

A qualitative model evaluation illustrated the validity of our modelling methodology (Fig. 4). Saturation trends where both spatially and temporally similar, which suggests that our root water uptake model was sufficiently accurate for predicting field scale dynamics under partially saturated conditions (Fig. 4 (a)). Similarly, our model was also able to capture the influence that the roots have on soil solution P concentrations (Fig. 4 (b)). While these estimates were more qualitative than the saturation comparison, the influence that roots have on taking up dissolved P impact the P distribution in the soil in a similar manner. The model did not predict the exact saturation magnitudes due to differences in soil physical properties, root distribution, and unknown lower boundary conditions. However, if needed, our model could be fit directly to the specific data, possibly allowing us for inverse modelling to estimate the soil physical properties and root suction pressures given information about the root distributions. Our model could likely be fit to the exact P profiles measured in these studies as well. However, this was outside of the scope of our study. The model validation grants us more confidence in our models ability to predict water and P uptake from field systems.

For the basic scenarios, the model was able to deal with early and late precipitation scenarios (Fig. 5). The dynamic differences considering the early (Fig. 5 (a)) vs late (Fig. 5 (b)) scenarios illustrate some differences in the dynamics of the soil moisture along the profile. Both profiles appear characteristically the same after 90 days. However, while the profiles are qualitatively the same, the water uptake from the two different scenarios differs drastically (Fig. 6). Though soil water content with depth under different rainfall scenarios may appear similar after 90 days, the preceding dynamics are very different. Information regarding the dynamics of soil water movement cannot be extracted from the steady state soil water content profile measured at 120 days, but must consider changes over time.

Between scenarios with root hairs vs no root hairs (Fig. 7 and Fig. 8), P profiles exhibit little difference in overall concentrations. The P concentrations down the soil depth remained largely unchanged*,* thus we only focus on the zone of the rooting region. The lack of change in the P concentrations along the soil depths below the rooting region can be attributed to several factors. Effective diffusivity of P in soil is extremely low, thus any movement of dissolved P mainly occurs via advection (Nye and Tinker 1977). However, P readily becomes bound to finer textured soil particles (Barber 1995). As a result, the fraction of dissolved P will likely be considerably less than the surface adsorbed P, with P_sorbed_:P_solution_ proportions ranging from 50:1 to 200:1 [mg kg^−1^_sorbed_: mg L^−1^_solution_] (Barber 1995).

Although P concentrations change marginally over a single growing season, simulation that were run for several growth cycles under realistic precipitation patterns revealed a noticeable decrease in soil P concentrations for soils containing plants with root hairs (Fig. 11). Most UK soils have abundant P reserves, but fertiliser is still applied as most P is adsorbed to soil and unavailable to plants. Root hairs clearly enhance the uptake of this P, leading to potentially greater nutrient use efficiency and decreased needs for fertiliser application. Taking barley as an example, which is grown on 1× 10^6^ ha of land in the UK, the 0.001 mol_p_ m^−3^ difference in P that we found between plants with and without root hairs (after six growing seasons), equates to over 150 t of P captured from the soil. The same area of land has 24,000-24,500 t of P added to it each year (DEFRA 2017), which amounts to 144,000-147,000 t of P over the 6 growing seasons. The total quantity of P taken up by the root hairs amounts to less than 0.1% of the P input. Thus, while root hairs enhance plant P acquisition, the effect of root hairs poses no risk of mining soils under current production practices.

Despite the limited changes of P concentrations down the soil profiles, the P uptake rates by the roots were significantly different under the precipitation conditions and the initial quantities of P in the soil (Fig. 9). The influence of the root hairs was modelled to account for 50% of the total P taken up by the plant root. This was in accordance with previous experimental literature (Brown et al. 2000; Keyes et al. 2013). These results appear consistent with modelled P uptake trends that only considered solute transport in soil (Itoh and Barber 1983). Image based modelling of root hair enhanced P-uptake also estimated that root hairs account for up to 50% the total P uptake (Daly et al. 2016). Our field scale model results are similar to these image based results. The P uptake rate by the roots scaled by 70% as a result of soil P concentrations reduction by 70% (comparing Fig. 9 (a-b) to (c-d)). This is expected, as the root uptake rates (Eqs. (26), (27), and (30)) are related to the P concentration in the soil via Michaelis-Menten kinetics (Nye and Tinker 1977). Furthermore, this is consistent with previous experiments (Brown et al. 2012b). In glasshouse experiments that considered a soil with an initial inorganic P content of 590 mg_P_ kg_bulk_^−1^, the amount of P accumulated in the plant shoots nearly doubled after 500 mg_P_ kg_bulk_^−1^ was added to the soil (Brown et al. 2012b).

The simulations also revealed the sensitivity of the uptake rates to the precipitation events (Fig. 9 (a, c) vs (b, d)). Phosphorus uptake rates were suppressed by 25-60% if the onset of precipitation was delayed by 60 days. The results from this study appear more pronounced than those found in the image based modelling study (Daly et al. 2016), which only saw marginal changes in the nutrient uptake due to drier conditions. As our field scale model considers large spatial averages, we think that it is more likely that full roots (and root hairs) may be under complete drought conditions, while other roots may still have to access smaller wet subdomains, and these would spatially manifest in greater reductions in the overall root uptake. We also note that the image based modelling study (Daly et al. 2016) maintained fixed soil moisture for their different scenarios, which facilitated nutrient fluxes to the root hairs whilst in our study the soil water saturation was considered dynamic.

Similar experiments considering maize roots have seen similar reductions in the amount of P taken up by plants under variable drought conditions (Resnik 1970); consistent with our model results. The P-uptake theoretically peaks within the first 2 months under wet conditions (Fig. 9), illustrating that the soil water regime plays a considerable role in P use efficiency. While plant yields will likely depend on the quantity of plant available nutrients in the soil, our results are consistent with the claim that plant P use efficiency (in our case, the P uptake rates) is influenced by frequency of precipitation (Silber et al. 2003). Moist conditions during early stages of a growing season would increase plant P use efficiency.

There were various assumptions in the derivation of the model, which focused on the impact of root hairs on P capture and uptake. Overland flow was omitted from this study. Prolonged flooding is not modelled as barley can only survive several days of waterlogging. Future studies could investigate flooding by incorporating the potential-form of Richards’ equation as presented in Duncan et al. (2018), where an approach how to swap between flooded and non-flooded boundary conditions is described. Although evaporation influences the net water fluxes in the soil, we do not explicitly include this process, as the focus of the study is on root soil interactions. To include our model in a broader scale, it would be important to consider different climatic variables (i.e. temperature, relative humidity, and wind speed) in order to estimate surface evaporation as done in previous studies (Heppell et al. 2014). For our study site, where rainfall exceeds evaporation and there is a net eluviation of nutrients to deeper depths, this assumption is fair, but for drier regions it would need to be included.

While the model simplifies the effect that root hairs have under natural environmental conditions, it provides predictions that agree with experimental observations. For example, experimental results suggest that root hairs nearly alleviate the impacts of drought entirely (Brown et al. 2012b). While our model suggests a strong mitigation to the effects of drought (e.g. maintaining significantly higher P uptake rates with root hairs compared to no hair), drought still has a comparative effect on the uptake rates (Fig. 9 (a, c) vs (b, d)). The enhanced resistance to drought may be due to the modification that root hairs impart on the soil structure local to the root interface (Koebernick et al. 2017), thus augmenting the moisture dynamics and, therefore, nutrient fluxes in the rhizosphere. Future work could develop more detailed models that better account for local rhizosphere structures (Daly et al. 2017) in the context of nutrient fluxes and considering how root hairs impact water uptake (Carminati et al. 2017). Similar to how rhizosphere features have impacts on soil chemistry, local microbiota will also be affected by root exudates, which will likely create feedbacks and possible mutualistic benefits (Dupuy and Silk 2016; Kuzyakov and Blagodatskaya 2015). Future work could try to consider the ensemble of these various rhizosphere impacts on the pore scale and how they upscale to the field.

## Conclusions

- We developed a field scale model that considers the role that root hairs play in soil P acquisition and compares their relative impact to varying precipitation patterns and differing initial soil P quantities and distributions.
- Results show that for individual growing seasons, P concentrations down the soil profile do not appear to change significantly. However, noticeable changes can be detected over several growing periods. Differences between simulations with and without root hairs suggest that, while root hairs play an important role in obtaining P, they pose negligible risk to soil nutrient mining under current agricultural procedures.
- Although root hairs account for up to 50% of the total P taken up by the plant roots, increasing the P content by 70% increased the root P uptake rate by 64%. A delayed precipitation scenario reduced P uptake rate by as much as 60%. These three features all play a critical role in understanding plant roots P use efficiency.
- The model results illustrate the significance of soil moisture during the growth season and suggests that irrigation strategies could be employed during the first 2 months of a given growing season to optimize the P use efficiency.
- We note that all of the results from this study were based on model simulations, and we stress that more future field scale studies should be carried out to measure the impact of small scale rhizosphere features on field scale processes.

## Electronic supplementary material

ESM 1 (DOCX 40 kb)
