# Supplementary material for: Significance of root hairs at the field scale – modelling root water and phosphorus uptake under different field conditions
Source: Plant Soil. 2019 Dec 6;447(1):281–304. doi: 10.1007/s11104-019-04308-2 (PMC7062663; doi:10.1007/s11104-019-04308-2)
Supplement: Supplementary file 1 — (DOCX 40 kb) [file 11104_2019_4308_MOESM1_ESM.docx]

# Supplementary material 1: Full list of expressions

Table S3 List of expressions and variables

| Expression | Description | SI Units |
| --- | --- | --- |
| $a$ | Primary root radius | [m] |
| $b$ | Soil buffer power for Phosphate | [-] |
| $\beta$ | 1st order lateral branching angle | [rad] |
| $\tilde{c}$ | Phosphate concentration | [mol m^-3^_water_] |
| $\tilde{c}_{0}$ | Initial Phosphate concentration | [mol m^-3^_water_] |
| $c_{0}$ | Dimensionless Initial Phosphate concentration | [-] |
| $c$ | Dimensionless Phosphate concentration | [-] |
| $c_{0,c}$ | Dimensionless initial Phosphate distribution magnitude | [-] |
| $D$ | Unsaturated soil water diffusivity | [-] |
| $d$ | Nutrient diffusivity impedance | [-] |
| $D_{0}$ | Soil water diffusivity scaling factor | [m^2^_water_ s^-1^] |
| $\delta$ | Porosity over buffer power | [-] |
| $D_{f}$ | Nutrient diffusivity in free water | [m^2^_phosphate_ s^-1^] |
| $\epsilon$ | Non-dimensionalized saturated hydraulic conductivity | [-] |
| $\tilde{F}$ | Total volumetric P uptake | [mol m^-3^_soil_ s^-1^] |
| $F_{0}^{*}$ | Dimensionless volumetric P uptake by Primary root | [-] |
| $F_{1}^{*}$ | Dimensionless volumetric P uptake by lateral root | [-] |
| $F$ | Dimensionless total Volumetric P uptake | [-] |
| $F_{m}$ | Maximum rate of nutrient uptake for P | [mol m^-2^ s^-1^] |
| $F_{w}$ | Volumetric root water uptake | [m^3^_water_ m^-3^_soil_ s^-1^] |
| $F_{r}$ | Dimensionless volumetric P uptake neglecting root hairs | [-] |
| $F_{w}$ | Dimensionless root water uptake | [-] |
| $g$ | gravity | [m s^-2^] |
| $\gamma$ | Euler constant | [-] |
| $k$ | Unsaturated soil water permeability | [-] |
| $K$ | Unsaturated soil hydraulic conductivity | [-] |
| $l_{0,f}$ | Maximum length of primary root | [m] |
| $l_{1,f}$ | Maximum length of lateral root | [m] |
| $\kappa_{0}$ | Dimensionless primary root hydraulic conductivity | [-] |
| $\kappa_{00}$ | Dimensionless lateral root hydraulic conductivity | [-] |
| $\hat{k}$ | Normal vector in the vertical direction | [-] |
| $l_{i,f}$ | Maximum length of ith order root | [m] |
| $K_{m}$ | Michaelis-Menten coefficient | [mol m^-3^_water_] |
| $k_{r}$ | Radial root hydraulic conductivity | [m Pa^-1^ s^-1^] |
| $k_{s}$ | Saturated soil water permeability | [m^2^_pore_] |
| $K_{s}$ | Saturated soil hydraulic conductivity | [m^3^_water_ s^-1^ m^-2^_soil_] |
| $k_{z}$ | Xylem water conductivity | [m^4^ Pa^-1^ s^-1^] |
| $k_{z1}$ | Xylem water conductivity of the lateral root | [m^4^ Pa^-1^ s^-1^] |
| $l_{0}$ | Primary root length | [m] |
| $l_{0,a}$ | Branching depth | [m] |
| $l_{n,0}$ | Nodal distance of roots | [m] |
| $\lambda_{w}$ | Dimensionless root water uptake coefficient | [-] |
| $l_{i}$ | Length of ith order root | [m] |
| $l_{i0}$ | Initial length of ith order root | [m] |
| $l_{ia}$ | Branching depth of ith order root | [m] |
| $L_{p}$ | Depth of the domain | [m] |
| $\mu$ | Dynamic viscosity of water | [Pa s] |
| $\Omega_{r}$ | Root domain | [m] |
| $\Omega_{s}$ | Soil domain | [m] |
| $\tilde{p}_{a}$ | Atmospheric pressure | [Pa] |
| $\tilde{p}_{c}$ | Critical soil suction | [Pa] |
| $\phi$ | Difference between wilting point and zero tension moisture | [m^3^_water_ m^3^_bulk_] |
| $\tilde{p}_{r}$ | Root uptake pressure | [Pa] |
| $p_{r}$ | Dimensionless root pressure | [-] |
| $\psi_{1}$ | First order lateral distribution | [m^-1^] |
| $\psi_{H}$ | Root hair indicator function | [-] |
| $\tilde{p}$ | Soil matric suction | [Pa] |
| $p$ | Dimensionless soil suction | [-] |
| $q_{r}$ | Radial root water flux | [m^3^_water_ s^-1^ m^2^_root_] |
| $Q_{r1}$ | Volumetric water flow through lateral roots | [m^3^_water_ s^-1^] |
| $Q_{z}$ | Volumetric water flow through the Xylem | [m^3^_water_ s^-1^] |
| $R$ | Dimensionless P diffusivity coefficient | [-] |
| $\rho$ | Density of water | [kg_water_ m^-3^_water_] |
| $r_{i}$ | Root growth rate | [m s^-1^] |
| $S$ | Saturation degree | [-] |
| $S_{0}$ | Initial saturation | [-] |
| $\tilde{t}$ | time | [s] |
| $t$ | Dimensionless time | [-] |
| $\tilde{\boldsymbol{u}}$ | Volumetric water flux in soil | [m^3^_water_ s^-1^ m^-2^_soil_] |
| $\tilde{w}$ | Water flux at the surface | [m^3^_water_ s^-1^ m^-2^_soil_] |
| $w$ | Dimensionless water flux at the surface | [-] |
| $z$ | Soil depth | [m] |
| $\zeta$ | Initial nutrient decay | [-] |
| $\hat{\tilde{z}}$ | Branching depth | [m] |
| $z'$ | Integration variable | [m] |
| $z$ | Dimensionless soil depth | [-] |

# Supplementary material 2: Non-dimensionalized equations

Following Roose and Fowler (Roose and Fowler 2004a), we non-dimensionlize the model in order to couple eqs. (11) and (25). Considering the following scaling relations: $\tilde{z}=z/l_{0,f}$, $\tilde{t}=t(D_{0}/(bl_{0,f}^{2}))$, and $\tilde{c}=c/K_{m}$, where $K_{m}(=5.8\times{10}^{-3}$mol m^-3^ ) is the Michaelis-Menten parameter (Roose et al. 2001), we could infer that the non-dimensionalized eqs. (11) and (25) would become:

|  | $\left\{ \begin{aligned} \delta\frac{\partial S}{\partial t}=\frac{\partial}{\partial z}\boldsymbol{(}D\left( S \right)\frac{\partial S}{\partial z}-\epsilon K\left( S \right)\boldsymbol{)} -F_{w}, z\in\boldsymbol{\Omega}_{\mathbf{s}} \\ D\left( S \right)\frac{\partial S}{\partial z}-\epsilon K\left( S \right)= -w, z=0 \\ D\left( S \right)\frac{\partial S}{\partial z}-\epsilon K\left( S \right)= 0, z=L_{P}/l_{0,f} \\ S\left( 0,z \right)=S_{0}, t=0 \end{aligned} \right.$ | (S2. 1) |
| --- | --- | --- |

and:

|  | $\left\{ \begin{aligned} \left( 1+\delta S \right)\frac{\partial c}{\partial t}-\left( D\left( S \right)\frac{\partial S}{\partial z}-\epsilon K\left( S \right) \right)\frac{\partial c}{\partial z}=R\frac{\partial}{\partial z}\left( \left( S \right)^{d+1}\frac{\partial c}{\partial z} \right)-F+F_{w}c, z\in\boldsymbol{\Omega}_{\mathbf{s}} \\ RS^{d+1}\frac{\partial c}{\partial z}+\left( D\left( S \right)\frac{\partial S}{\partial z}-\epsilon K\left( S \right) \right)c= 0, z=0 \\ RS^{d+1}\frac{\partial c}{\partial z}+\left( D\left( S \right)\frac{\partial S}{\partial z}-\epsilon K\left( S \right) \right)c= 0, z=L_{P}/l_{0,f} \\ c\left( 0,z \right)=c_{0}(z), t=0 \end{aligned} \right.$ | (S2. 2) |
| --- | --- | --- |

where the dimensionless coefficient of the time-derivative is defined as $\delta=\phi/b$ , the dimensionless saturated hydraulic conductivity becomes $\epsilon=K_{s}l_{0,f}/D_{0}$, and the dimensionless nutrient diffusivity is now $R=D_{f}\phi^{d+1}/D_{0}$. Furthermore, the scaled domain $\boldsymbol{\Omega}_{\mathbf{s}}=[0,\frac{L_{p}}{l_{0,f}})$. The precipitation/irrigation is defined as $w=\tilde{w} l_{0,f}/D_{0}$. The sink term in eq. (S2. 1) is now defined as:

|  | $F_{w}=\frac{l_{0,f}^{2}}{D_{0}}\tilde{F}_{w}=\lambda_{w}\left( p\left( S \right)-p_{r} \right)$ | (S2. 3) |
| --- | --- | --- |

where $p_{r}=\tilde{p}_{r}/|P|$ is the dimensionless water pressure in the root, $p=\tilde{p}/|P|$ is the dimensionless water pressure in the soil, $|P|$ (=3$\times$10^5^ Pa) is the assumed maximum root pressure at the base, and the coefficient $\lambda_{w}$ represents:

|  | $\lambda_{w}=\left( \frac{l_{0,f}^{2}}{D_{0}} \right)\frac{2\pi ak_{r}+\sqrt{2\pi ak_{r}k_{z,1}}\psi_{1}\left( z \right)}{\pi\left( a+l_{1,f}\cos\left( \beta\right) \right)^{2}}\vert P\vert$ | (S2. 4) |
| --- | --- | --- |

and the dimensionless root pressure equations become:

|  | $\left\{ \begin{aligned} -\frac{\partial^{2}p_{r}}{\partial z^{2}}=\boldsymbol{(}\kappa_{0}^{2}\boldsymbol{+}\kappa_{00}^{2}\psi_{1}(z)\boldsymbol{)}\left( p-p_{r} \right), z\in\boldsymbol{\Omega}_{\boldsymbol{r}} \\ \frac{\partial p_{̃r}}{\partial z}=0, z=l_{0}/l_{0,f} \\ p_{r}=-1, z=0 \end{aligned} \right.$ | (S2. 5) |
| --- | --- | --- |

where ${\frac{k_{z}}{l_{0,f}^{2}}\kappa}_{0}^{2}$ and $\frac{k_{z}}{l_{0,f}^{2}}\kappa_{00}^{2}$ are the coefficients in the dimensional version (eq. (19)). The dimensionless nutrient uptake term is broken up into the contributions by the primary and lateral roots:

|  | $F=F_{0}^{*}+F_{1}^{*}$ | (S2. 6) |
| --- | --- | --- |

Where $F_{0}^{*}$ and $F_{1}^{*}$ are the dimensionless volumetric nutrient uptake rates by the primary and lateral roots respectively. For the primary roots, the uptake rate derived from the matched asymptotic solution of the radial uptake flux (Roose et al. 2001) is defined by (Roose and Fowler 2004a):

|  | $F_{0}^{*}=\frac{2\Lambda_{0}c}{1+c+L_{0}\left( z,t \right)+\sqrt{4c+\left( 1-c+L_{0}\left( z,t \right) \right)^{2}}}$ | (S2. 7) |
| --- | --- | --- |

with $\Lambda_{0}$ is defined as:

|  | $\Lambda_{0}=\frac{2aF_{m}l_{0,f}^{2}}{\left( a+l_{1,f}\cos\left( \beta\right) \right)^{2}K_{m}D_{0}}$ | (S2. 8) |
| --- | --- | --- |

where $F_{m}$(=3.26$\times$10^-8^ mol m^-2^ s^-1^) is the maximum rate of root nutrient uptake for P, $L_{0}$ is defined as:

|  | $L_{0}\left( z,t \right)=\frac{\lambda_{0}}{2 S^{d+1}}\ln\left( \left( \alpha_{0}t+\alpha_{00}\ln\left( 1-z \right) \right)\left( \frac{S^{d+1}}{1+\delta S} \right)+1 \right)$ | (S2. 9) |
| --- | --- | --- |

where $\lambda_{0}=F_{m}a/(D_{f}\phi^{d+1}K_{m})$, $\alpha_{0}=4e^{-\gamma}\left( \frac{D_{f}\phi^{d+1}}{a^{2}b} \right)\left( \frac{bl_{0,f}^{2}}{D_{0}} \right)$, $\gamma\approx$ 0.5772 is the Euler-Mascheroni constant (Lagarias 2013; Roose and Fowler 2004a), $\alpha_{00}=4e^{-\gamma}\left( \frac{D_{f}\phi^{d+1}}{a^{2}b} \right)\left( \frac{l_{0,f}}{r_{0}} \right)$, where $r_{0}$ [m s^-1^] is the maximum primary root growth rate. For the uptake by the first lateral roots, the model considers the summation of the uptake by all of the lateral roots in the branching zone:

|  | $F_{1}^{*}=\int_{\hat{z}}^{z} \frac{2\Lambda_{1}c \psi_{1}(z')dz'}{1+c+L_{1}\left( z,t;z' \right)+\sqrt{4c+\left( 1-c+L_{1}\left( z,t;z' \right) \right)^{2}}}$ | (S2. 10) |
| --- | --- | --- |

where:

|  | $\Lambda_{1}=\frac{2aF_{m}l_{0,f}^{3}}{{\cos\left( \beta\right)\left( a+l_{1,f}\cos\left( \beta\right) \right)}^{2}K_{m}D_{0}l_{n,0}}$ | (S2. 11) |
| --- | --- | --- |

where $l_{n,0}$[m] is the nodal distance of lateral roots from one another. Similar to $L_{0}$, $L_{1}$ is defined as:

|  | $L_{1}\left( z,t;z' \right)=\frac{\lambda_{1}}{2 S^{d+1}}\ln\left( \left( \alpha_{1}t+\alpha_{11}\ln\left( 1-\left( z^{'}+l_{a,0} \right) \right)+\alpha_{111}\ln\left( 1-\frac{z-z^{'}}{l_{1,f}\cos\left( \beta\right)} \right) \right)\left( \frac{S^{d+1}}{1+\delta S} \right)+1 \right)\ln\left( \left( \alpha_{1}t+\alpha_{11}\ln\left( 1-\left( z^{'}+l_{a,0} \right) \right)+\alpha_{111}\ln\left( 1-\frac{z-z^{'}}{l_{1,f}\cos\left( \beta\right)} \right) \right)\left( \frac{S^{d+1}}{1+\delta S} \right)+1 \right)$ | (S2. 12*)* |
| --- | --- | --- |

where $\lambda_{1}=F_{m}a_{1}/(D_{f}\phi^{d+1}K_{m})$, where $a_{1}$[m] is the radius of the first order roots, $\alpha_{1}=4e^{-\gamma}\left( \frac{D_{f}\phi^{d+1}}{a_{1}^{2}b} \right)\left( \frac{bl_{0,f}^{2}}{D_{0}} \right)$, $\alpha_{11}=4e^{-\gamma}\left( \frac{D_{f}\phi^{d+1}}{a_{1}^{2}b} \right)\left( \frac{l_{0,f}}{r_{0}} \right)$, and $\alpha_{111}=4e^{-\gamma}\left( \frac{D_{f}\phi^{d+1}}{a_{1}^{2}b} \right)\left( \frac{l_{1,f}}{r_{1}} \right)$ where $r_{1}$ [m s^-1^] is the maximum lateral root growth rate determined based on the ratio between $\alpha_{11}/\alpha_{111}$ and table 2 in Roose and Fowler (Roose and Fowler 2004a). As the computed values for $\alpha_{11}$ and $\alpha_{111}$ are relatively low compared to $\alpha_{1}$, the formulation was simplified by solely considering $\alpha_{1}$. We note that there was very little change in the lateral root uptake rate, and the simulations ran more stable.
As in the dimensional equations, the solute uptake by lateral roots requires that the zone of influence is solved for a given root in the domain $(\hat{z}, z]$ (Fig 2). For any given branching point $\hat{z}$, the lateral root that branches from the primary root has zone of influence at a range of given depths (Fig 2) defined as:

|  | $z= \hat{z}+\frac{l_{1,f}}{l_{0,f}}\cos\left( \beta\right)\left( 1-e^{-\frac{r_{1}t}{l_{1,f}}}\left( 1-\left( \hat{z}+\frac{l_{a,0}}{l_{0,f}} \right)^{-\frac{r_{1}l_{0,f}}{r_{0}l_{1,f}}t} \right) \right)$ | (S2. 13) |
| --- | --- | --- |

thus for any $z$ at any point in time, $\hat{z}$ has to be numerically computed. Parameters used for modelling could be found in Table 1.
